# Multi-method brain imaging reveals impaired representations of number as well as altered connectivity in adults with dyscalculia

**DOI:** 10.1016/j.neuroimage.2018.06.012

**Published:** 2019-04-15

**Authors:** Jessica Bulthé, Jellina Prinsen, Jolijn Vanderauwera, Stefanie Duyck, Nicky Daniels, Céline R. Gillebert, Dante Mantini, Hans P. Op de Beeck, Bert De Smedt

**Affiliations:** a Brain and Cognition, Faculty of Psychology and Educational Sciences, KU Leuven, Leuven, 3000, Belgium; b Neuromotor Rehabilitation, Biomedical Sciences, KU Leuven, Leuven, 3000, Belgium; c Parenting and Special Education Research Unit, Faculty of Psychology and Educational Sciences, KU Leuven, Leuven, 3000, Belgium; d Experimental Psychology, University of Oxford, Oxford, OX1 3UD, UK; e Research Center for Motor Control and Neuroplasticity, KU Leuven, Leuven, 3001, Belgium; f Neural Control of Movement Laboratory, ETH Zurich, Zurich, 8057, Switzerland

## Abstract

Two hypotheses have been proposed about the etiology of neurodevelopmental learning disorders, such as dyslexia and dyscalculia: representation impairments and disrupted access to representations. We implemented a multi-method brain imaging approach to directly investigate these representation and access hypotheses in dyscalculia, a highly prevalent but understudied neurodevelopmental disorder in learning to calculate. We combined several magnetic resonance imaging methods and analyses, including univariate and multivariate analyses, functional and structural connectivity. Our sample comprised 24 adults with dyscalculia and 24 carefully matched controls. Results showed a clear deficit in the non-symbolic magnitude representations in parietal, temporal and frontal regions, as well as hyper-connectivity in visual brain regions in adults with dyscalculia. Dyscalculia in adults was thereby related to both impaired number representations and altered connectivity in the brain. We conclude that dyscalculia is related to impaired number representations as well as altered access to these representations.

## Highlights

• Dyscalculia affects 5–7% of population and is as prevalent as dyslexia.
• We applied wide variety of neuroimaging techniques to investigate this hypothesis.
• First neuroimaging study with adults diagnosed with dyscalculia.
• Impaired non-symbolic number representations are observed across cortex.
• Increased functional connectivity between temporo-occipital regions in dyscalculia.

## Introduction

In neuroscience, hypotheses about the neural basis of disorders often fall into two categories: dysfunction in specific brain regions and cognitive representations versus disrupted connectivity and impaired access to these representations. This distinction has been present in the history of neuroscience and started when the localized function approach in language disorders was upended by the concept of disconnection syndromes, as introduced by Wernicke (Spreen et al., 1995). A similar distinction has been suggested for the etiology of neurodevelopmental learning disorders, such as dyslexia (Boets et al., 2013) and dyscalculia (Price and Ansari, 2013).

Recently, a multi-method brain imaging approach was used in adults with dyslexia to directly compare the representation versus access hypotheses and this approach combined measures of the quality of neural representations with measures of connectivity (Boets et al., 2013). The present study applied the same approach to another neurodevelopmental learning disorder, i.e. dyscalculia, which is a disorder in learning to calculate. We used a similar multi-method brain imaging approach as in Boets et al. (2013) to investigate whether impaired representations, impaired access or both (the representation and access hypotheses are not mutually exclusive) explain the origin of dyscalculia.

Dyscalculia is far less frequently investigated compared with dyslexia or autism spectrum disorder (Bishop, 2010), yet it is as prevalent as these disorders (Butterworth et al., 2011; Elsabbagh et al., 2012) and it affects about 5–6% individuals of the population (Rubinsten and Henik, 2009). Dyscalculia is thought to originate from impaired numerical magnitude processing (De Smedt et al., 2013). However, to date no study investigated whether the neural magnitude representations and the access to these representations are impaired in adults with dyscalculia in one sample with the same task. The investigation of the origin of these impairments is also of practical relevance, because lower levels of numerical abilities have been associated with lower income (Estrada-Mejia et al., 2016) and socio-economic status (Ritchie and Bates, 2013), poorer medical decision making (Reyna et al., 2009), and even greater likelihood of mortgage default (Gerardi et al., 2013).

**Fig. 1:**
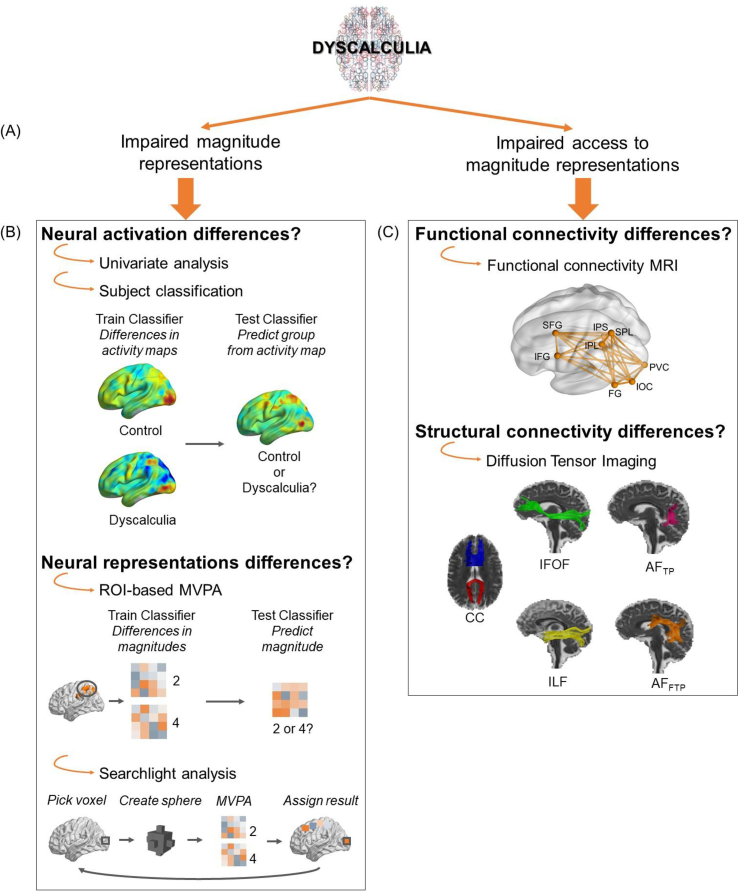
Overview of the current study. Two hypotheses about the etiology of dyscalculia (*A*), and the main approaches to test them (*B-C*). **Univariate fMRI analyses** test if there is a significant difference in *brain activation* between the two groups. **Multivariate fMRI analyses (MVPA)** use machine learning algorithms to examine *activation patterns* at the whole brain level (subject classification), region of interests level (ROI-based MVPA), or in small regions across the whole brain (searchlight analysis). **Subject classification** examines whether the *whole brain activation patterns* of both groups are different enough to correctly classify an individual as a person with or without dyscalculia. **ROI-based MVPA** allows one to investigate if the activation patterns of numerical magnitudes in ROIs differ in quality between individuals with and without dyscalculia. **Searchlight analyses** search throughout the entire brain where local differences in the quality of numerical magnitude activation patterns differ between the two groups. **Functional connectivity analyses** examine where in the brain the communication between grey matter areas differs between individuals with and without dyscalculia. **Structural connectivity analyses** investigate the differences in white matter connectivity between the two groups. Abbreviations: Regions of Interest (ROI), multivoxel pattern analysis (MVPA), magnetic resonance imaging (MRI), superior and inferior frontal gyrus (SFG, IFG), intraparietal sulcus (IPS), inferior parietal lobule (IPL, SPL), fusiform gyrus (FG), inferior occipital cortex (IOC), primary visual cortex (PVC), corpus callosum (CC), inferior fronto-occipital fasciculus (IFOF), inferior longitudinal fasciculus (ILF), tempo-parietal arcuate fasciculus (AF_TP_), and fronto-temporoparietal arcuate fasciculus (AF_FTP_).

The available neuroimaging studies on dyscalculia, which mainly comprised studies with children, mainly considered the overall activation level in cortical regions associated with number processing and restricted their focus to the intraparietal sulcus (IPS). These studies have yielded mixed results and, depending upon task requirements, hypo-activation (Ashkenazi et al., 2012; Mussolin et al., 2010b; Price et al., 2007) as well as hyper-activation (Rosenberg-Lee et al., 2015; Simos et al., 2008) in the IPS in children with dyscalculia compared with matched controls have been reported. These differences in brain activity suggest an altered task-modulation of the IPS during number processing, but they do not directly provide any information about the quality of the involved representations. The present study applied multivariate fMRI analyses to compare the quality of the number representations between adults with and without dyscalculia. We therefore can directly investigate whether the impaired neural number representations hypothesis holds in adults with dyscalculia (Fig. 1b).

It also has been suggested that the number representations themselves are not impaired in dyscalculia, but that these representations are difficult to access (Noël and Rousselle, 2011) (Fig. 1a). This is supported by behavioral studies showing that children with dyscalculia were only impaired in the processing symbolic but not non-symbolic magnitudes (De Smedt and Gilmore, 2011; Rousselle and Noël, 2007). At the neural level, there is some evidence for impaired access and altered connectivity. More specifically, children with dyscalculia have reduced white matter volume in right temporal-parietal cortex (Rykhlevskaia et al., 2009; see also Matejko and Ansari, 2015), and hyper-connectivity between the IPS and lateral fronto-parietal regions (Rosenberg-Lee et al., 2015), and between the IPS and areas of the default mode network (Jolles et al., 2016) has been reported in dyscalculia.

The current study is the first to directly test and compare both the hypothesis of impaired neural magnitude representations in dyscalculia and the hypothesis of impaired access to these neural representations. As a first step, we applied univariate fMRI analyses to investigate differences in activation levels between adults with and without dyscalculia. This allowed us to compare our data with previous fMRI studies of dyscalculia, which only used univariate analyses (Fig. 1b). We then extended the spectrum of activation related analyses by additionally including subject classification methods. Subject classification methods allowed us to directly verify the functional differences between the two groups that were strong enough to identify individuals with dyscalculia based on their brain activity only. As a second step, multivariate fMRI analyses were performed to directly test the hypothesis of impaired number representations (Fig. 1b). Third, connectivity analyses were ran to test the hypothesis of access impairments in dyscalculia (Fig. 1c). We used the same regions of interest (ROIs) in both the multivariate fMRI analyses and in the functional connectivity analyses. It should be noted that the impaired number representations hypothesis and impaired access to number representations hypothesis are not mutually exclusive. Both hypotheses could be either correct or incorrect.

## Methods

### Participants

Participants were 54 adults, who took part in this study as paid volunteers. Due to technical issues with the MR scanner, useful data were only acquired for 48 participants (all females, aged between 18 and 27, three left-handed participants with dyscalculia, and two left-handed participants in the control group). This resulted in a final sample of 24 participants with dyscalculia and 24 control participants with normal mathematics achievement. All participants had normal or corrected-to-normal vision and they all reported no neurological or psychiatric history. Prior to participating in the study, each participant was interviewed to confirm that the participants with dyscalculia, and none of the control participants, met the DSM-V criteria for “Specific Learning Disorder with Impairment in Mathematics”. These criteria comprised: (a) difficulties in mastering number sense, number facts, or calculation for at least 6 months (b) difficulties with mathematical reasoning (i.e. difficulties in applying mathematical concepts, facts or procedures to solve quantitative problems); for at least 6 months; (c) affected academic skills that were substantially and quantifiably below those expected for the individual's chronological age, and that caused significant interference with academic performance or with activities of daily living; (d) a documented history of learning difficulties in mathematics during the school-age years; and (e) the learning difficulties were not better accounted for by intellectual disabilities, uncorrected visual or auditory acuity, other mental or neurological disorders, psychosocial adversity, lack of proficiency in the language of academic instruction, or inadequate education instruction (American Psychiatric Association, 2013, p. 67).

All participants provided two written informed consents, one before the behavioral session and one prior to scanning. The study was approved by the medical ethics committee of UZ/KU Leuven. None of the participants participated in previous neuroimaging studies of our research group (Bulthé et al., 2015, 2014a).

### Matching the dyscalculia and control groups

All participants successfully completed secondary school and they all were enrolled in higher education. The two groups were pairwise individually matched for their education in secondary school and college/university, sex, and age (Table 1). We evaluated their arithmetic and reading skills, motor speed, and intelligence to match the two groups for all measures, except for arithmetic. Statistical analyses for all the behavioral measures were done in Matlab (The MathWorks, Natrick, MA, version 8.3.0.532 (R2014a)).

**Table 1:** Descriptive statistics on matching variables.

	Dyscalculia	Control	*t[46]*	*p*	*Cohen's d*
**Descriptive information**					
N	24	24	–	–	–
Age (in years)	21.96 (2.16)	21.67 (2.20)	0.46	0.65	0.13
**Mathematical abilities**					
French Kit	35.00 (7.97)	54.50 (15.86)	5.38	<0.0001	−1.55
Tempo Test Arithmetic	113 (17.46)	148.75 (21.92)	6.25	<0.0001	−1.80
Arithmetic (WAIS)^a^	7.50 (2.06)	10.79 (2.21)	5.34	<0.0001	−1.54
**Reading**					
Z-score Reading	−0.20 (1.12)	0.20 (0.62)	1.49	0.14	−0.44
**IQ measures**					
Nonverbal– Matrix reasoning (WAIS) a	9.08 (2.90)	10.23 (3.01)	1.32	0.19	−0.39
Verbal – Vocabulary (WAIS) a	10.79 (2.99)	12.08 (2.26)	1.69	0.10	−0.48
**Motor speed task**					
Accuracy (% correct)	96.88 (10.82)	98.75 (2.21)	0.83	0.41	−0.24
Reaction time (s)	0.40 (0.08)	0.36 (0.06)	−1.80	0.08	0.57

NoteStandard deviations are shown in parentheses.

a Standardized score with *M* = 10 and *SD* = 3.

Mathematical abilities were assessed by three tests. The Tempo Test Calculation (De Vos, 1992) and French Kit (French et al., 1963) are two standardized paper-and-pencil tests for single- and multi-digit calculation (addition, subtraction, multiplication, and division) under time pressure. The arithmetic subtest of the WAIS (Wechsler Adult Intelligence Scale III), which involves the solution of verbally presented word problems without time pressure, was additionally administered. Reading abilities were assessed by reading as many real words (Brus, 1999) and pseudo-words (Van den Bos et al., 1994) as possible in 1 min. These two standardized reading tests were done to verify the absence of comorbidity with dyslexia. Verbal and non-verbal intelligence were investigated with the Vocabulary and Matrix Reasoning subtests of the WAIS, respectively. To verify whether any group differences in reaction time for behavioral mathematics tests or number processing tasks in the scanner were not due to group differences in processing speed, participants performed a motor speed reaction time task on a computer. During this task participants had to decide, in a rapid but accurate manner, at which side of the screen a white colored stimulus was presented, by pressing the corresponding key (De Smedt and Boets, 2010).

### fMRI data acquisition and analyses

#### Stimuli

The stimuli and design in this experiment were the same as in a previous study by Bulthé, De Smedt and Op de Beeck, 2014a. Stimuli in the experimental runs consisted of the numerical magnitudes 2, 4, 6 or 8, displayed as either symbolic numbers or collections of white dots on a black background (non-symbolic numbers). Similar to previously used methods (Dehaene et al., 2005), stimuli within each condition were varied in intensive (individual item size and inter-item spacing) and extensive (total luminance and total area spanned by the non-symbolic numbers) confounding parameters, and in addition we varied the position of the stimuli randomly across the displays. The adaptation of the symbolic numbers was minimized by varying their position and size across trials. Stimuli were presented with Psychtoolbox 3 (Brainard, 1997) in Matlab and projected via a NEC projector onto a screen located approximately 46 cm from participants' eyes.

#### Design

**Fig. 2:**
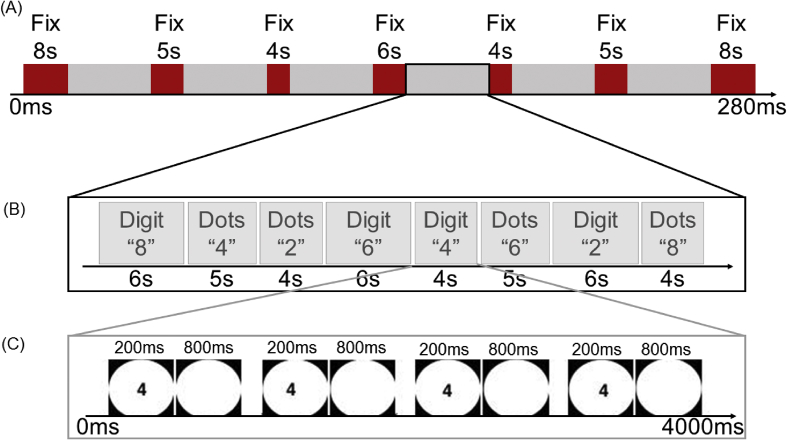
Summary of the experimental design; (a) Example of an experimental run; (b) Example of 8 experimental blocks; (c) Example of 4 trials within one experimental block.

The experimental runs had a short-block design with variable block duration and this design was the same as in Bulthé et al. (2014a). Short blocks (4, 5 or 6s) were used to prevent the loss of attention and to minimize potential adaptation effects during a single condition (Fig. 2). Each run consisted of 48 experimental blocks (each condition was presented in 6 blocks) and 7 fixation blocks (baseline). Two fixation blocks were presented for 8 s at the beginning and at the end of each run. The remaining five fixation blocks lasted 4, 5 or 6 s and they were presented after each 8th experimental block. In one experimental block, the same numerosity was repeated in sequences of 4, 5 or 6 trials in the same format. Participants had to perform a number comparison task in the experimental runs. We selected this task because it allowed us to explicitly access the numerical magnitude representations (Piazza et al., 2004; Pinel et al., 2004; Zorzi et al., 2011). Participants were instructed to evaluate whether the presented number was smaller (response with left index finger) or larger (response with right index finger) than five whenever the displayed format or numerosity changed.

Statistical analyses for accuracy and reaction time of the number comparison task in the scanner were done in Matlab. Each trial lasted for 1000 ms, during which a random example of the number was shown for 200 ms, followed by a fixation cross for 800 ms. One run lasted for 280 s and 8 to 12 experimental runs were acquired per participant. There was no significant difference in number of experimental runs acquired in the dyscalculia group (*M* = 11.83) and the control group (*M* = 11.95) (*t*_46_ = 0.69, *p* = 0.50).

The localizer runs consisted of the same localizer task as in Bulthé et al. (2015, 2014a). Participants had to subtract two numbers that ranged between 1 and 20 and they had to indicate whether the solution was even or odd. Answers were given via left or right keypresses and these were counterbalanced within each run. In each trial the subtraction problem was presented for 1700 ms, followed by a fixation cross for 300 ms. Two localizer runs were obtained per participant.

#### fMRI data acquisition

FMRI data were acquired in a 3T Philips Ingenia CX Scanner with a 32-channel head coil using a T2*-weighted echo-planar (EPI) pulse sequence (50 slices, 2.10 × 2.15 mm in plane acquisition voxel size, slice thickness 2 mm, interslice gap 0.2 mm, repetition time (TR) = 3000 ms, echo time (TE) = 30 ms, flip angle = 90, 100 × 97 acquisition matrix). For each participant, a T1-weighted anatomical volume was collected (182 slices, resolution 0.98 × 0.98 × 1.2 mm, TR = 9.6 ms, TE = 4.6 ms, 256 × 256 acquisition matrix).

#### fMRI preprocessing

The data were processed with the Statistical Parametric Mapping software (SPM 12, Wellcome Department of Cognitive Neurology, London) in Matlab. Anatomical images were normalized to the standard brain template defined by the Montreal Neurological 152-brains average. Functional images were corrected for slice timing differences. Realignment between images was performed to correct for motion across and within sessions and this resulted in a set of motion parameters that were used as confounds when modelling the BOLD response via a general linear model. None of the collected experimental runs had to be excluded for extensive motion (based on the criterion of movement in any direction of more than one voxel size). Co-registration of the functional data and the anatomical image was performed. During normalization, functional images were resampled to a voxel size of 2 × 2 × 2 mm. Additionally, functional images were spatially smoothed to suppress high-frequency noise. The images were convolved with a Gaussian kernel of 4 mm full-width at half maximum (FWHM) for subsequent multivariate voxel pattern analyses (see Op de Beeck, 2010), while 8 mm FWHM was used for subsequent second-level univariate analyses.

#### Statistical analysis

For each voxel, the experimental effect in a block was estimated by applying a general linear model. This resulted in beta-values for each condition (including the fixation condition) and six motion parameters per run (the x, y, and z values for both rotation and translation). *T*-statistics (resulting from conditions vs. baseline) were estimated and used as input for subsequent multivariate analysis. This was done because *t-*statistics take both the mean and the variance of the activations into account (Misaki et al., 2010).

#### ROIs

We selected ROIs that were activated during number comparison tasks in previous studies (Bulthé et al., 2015, 2014a; Holloway et al., 2013; Lyons and Ansari, 2009; Piazza et al., 2007). We also included ROIs in the occipital and temporal cortex that are often studied in other relevant research domains (e.g., visual perception and reading), but that are not specifically associated with numerical processing (Burr and Ross, 2008; Saygin et al., 2016; Walther et al., 2009).

The 17 ROIs were tested in a hierarchical order as introduced in Bulthé et al. (2014a) (Fig. 2). This approach combines the strength of global large-scale volumes to pick up highly distributed effects with the strength of smaller local ROIs to pick up more focal differences (Bulthé et al., 2014b). Four spatial scales were introduced: whole cerebral cortex (“All Regions ROI”), lobes, regions, and sub-regions. At the first level, (I) All Regions (all grey matter voxels with significantly increased activity in the task vs. baseline contrast in the localizer task in a participant), we investigated group differences across all grey matter voxels. If (and only if) there was a significant group difference at a higher level, the group comparison was tested at the lower level. *P*-values were FDR corrected across that level. Next (II), the 4 lobes were examined: frontal cortex, parietal cortex, temporal cortex, occipital cortex, and then (III), the Specific regions: fusiform gyrus (FG), inferior frontal gyrus (IFG), superior frontal gyrus (SFG), inferior occipital cortex (IOC), superior occipital gyrus, primary visual cortex (PVC), supramarginal gyrus, angular gyrus, inferior parietal lobule (IPL), superior parietal lobule (SPL), IPS. Finally, (IV) sub-regions within the IPS were tested: left anterior IPS (IPS_LA_), right anterior IPS (IPS_RA_), left posterior IPS (IPS_LP_), and right posterior IPS (IPS_RP_), were investigated.

All ROIs were created at the individual level, based on functional activity in the localizer scan, because this approach avoids the inclusion of voxels without any modulation by task or stimuli. From this point of view, this functional criterion serves as a feature selection, which is very often done in one way or another in multi-voxel decoding studies (Norman et al., 2006). For the ROIs for which an anatomical mask was available in the WFU PickAtlas Toolbox (Wake Forrest University PickAtlas, fmri. wfubmc.edu/cms/software), we selected those voxels from the anatomical mask that also showed increased activity (task minus fixation) during the independent localizer scans at an uncorrected threshold of *p* < 0.001. The full list of the ROIs made by the overlap between the anatomical mask and localizer data were as follows: Occipital Cortex, Temporal Cortex, Frontal Cortex, Parietal Cortex, Primary Visual Cortex, Inferior Occipital Cortex, Fusiform Gyrus, Inferior Frontal Gyrus, Superior Frontal Gyrus, Inferior Parietal Lobule and Superior Parietal Lobule. For the IPS and its subdivisions, we delineated these ROIs manually on the functional contrast of the localizer scans (uncorrected threshold at *p* < 0.01). The delineation of the IPS was carried out as we have done in previous studies (Bulthé et al., 2014a, 2015). The current approach thus provides the needed consistency between the present study and these related studies. More specifically, the borders of the IPS ROIs were determined on the basis of anatomical coordinates (Left IPS: MNI [-42 -42 42] to [-23 -62 51] and Right IPS: MNI [42−42 45] to [27–60 51]). The Anterior part was determined by the 4 most anterior slices, and the posterior part was determined by the 4 most posterior slices. This was done in a consistent manner across subjects. There was no significant difference between the groups in the number of voxels that were included in the ROIs (all *p*_uncorrected_ > 0.08; all *p*_FDR_ > 0.80).

The supramarginal gyrus, superior occipital gyrus, and angular gyrus were excluded from further analyses because they could not be defined in at least five subjects. This was because there were no voxels in the anatomical mask that showed increased activity during the localizer task. If it was not possible to identify one of the remaining ROIs in a participant, this participant was excluded in subsequent analyses for that particular ROI.

#### Correction for multiple comparisons

We applied the same hierarchical FDR-correction as in our previous work (Bulthé et al., 2014a). All group differences, for univariate and multivariate ROI-based analyses, were evaluated with a two-sided two-sample *t-*test. Within each level of the hierarchy, a correction for multiple comparisons (FDR) across all ROIs in that level was applied. This reasoning is similar to the well-known statistical approach of only testing a priori *t*-contrasts (e.g., pairwise comparisons) if an *F*-test that includes all conditions is significant. To be certain that we did not overlook any important smaller ROIs (i.e., in case a higher level ROI did not show a significant group difference), we also applied an FDR-correction across all ROIs. However, none of the ROIs survived this more stringent FDR-correction when the higher-level ROI did not show an effect.

#### Univariate analysis

For every participant, two contrasts for the experimental runs were calculated: symbolic numbers minus fixation and non-symbolic numbers minus fixation. For this analysis, no distinction was made between the numerical magnitudes within each format. A second-level group analysis in SPM12 was done for these contrasts to test activation differences in symbolic and non-symbolic numbers between the two groups at the whole brain level (threshold of *p* < 0.05 after FWE correction at voxel-wise level). The figures for this analysis were made with BrainNet Viewer (Xia et al., 2013). An ROI-based univariate analysis was also conducted to test for group differences within each format. We performed two-sample *t*-tests and corrected for multiple comparisons in a hierarchical manner, as described above.

#### Multivariate analysis

##### Subject classification based upon spatial variation in univariate activity levels

We used the functional contrasts ‘symbolic numbers minus fixation’ and ‘non-symbolic numbers minus fixation’ from the experimental runs for every participant. It is important to point out that this analysis differs from searchlight-based or ROI-based MVPA, as it does not investigate the underlying differences in the quality of the neural representations of symbolic numbers and non-symbolic numbers between the groups. Instead, it evaluates whether there is a more general difference in activation between the two groups when symbolic and non-symbolic numbers are processed. In this way, this analysis is more closely related to a second level univariate analysis than to multivariate ROI-based decoding and searchlight analyses. The main difference with a second level univariate analysis is that there is no activity-based comparison at the level of single voxels, but a spatial pattern comparison between the two groups across the whole brain or across a selected ROI. A similar subject classification approach has been used in research on other neurodevelopmental disorders, such as autism spectrum disorders (Uddin et al. (2011); Iuculano et al. (2014)) and dyslexia (Tamboer et al., 2016).

The subject classification analysis was implemented with custom code written in Matlab with the LIBSVM toolbox (Chang and Lin, 2011) that we made freely available via the Open Science Framework at https://osf.io/4rfz6/files/. The classification was performed with linear support vector machines with the following parameters (and these are the default values of the LIBSVM toolbox): a radial basis function kernel as decision function with parameter gamma set to 1; a C-SVC classification algorithm with parameter C set to 1. An intuitive explanation of the meaning of these parameters can be found at http://scikit-learn.org/stable/auto_examples/svm/plot_rbf_parameters.html. We applied a leave-one-pair-out-cross-validation (LPOCV) technique, similar to the procedure used by Ung et al. (2014). We opted for the LPOCV procedure because if we would apply a leave-one-out cross-validation there would be a “class imbalance”, i.e., there would be an unequal number of exemplars in the two groups during the classification, which would result in a bias to the largest group. With the LPOCV method, the classifier was trained on all participants, except for one random participant from the control group and one random participant from the dyscalculia group. Afterwards, the trained model was tested on this left-out pair. This procedure was repeated until each participant was left out once. Because of this random division into pairs, slightly different accuracies can occur depending on the division. The LPOCV was therefore ran 1000 times, and the results were averaged across all these repeats.

Statistics were obtained by a Monte Carlo Permutation test (Mourão-Miranda et al., 2005). The class labels of the training set were randomly permuted 1000 times and the same LPOCV procedure as described above was applied. A *p*-value for the subject classification accuracy was obtained by the number of times the permutation accuracy was greater than or equal to the subject classification accuracy, divided by 1000. For this subject classification, we noted that the permutation-based threshold for statistical significance was very similar to the threshold that would have been set by a simple parametric binomial test, which would take into account the proportion of participants classified in a particular group. To correct for multiple comparisons, we applied an FDR-correction across the four lobes and across the IPS regions.

##### Whole brain searchlight analysis of the decoding of neural magnitude representations

A whole brain searchlight analysis is particularly suited for finding where in the brain the local spatial activity pattern differs across conditions without selecting any ROIs (Kriegeskorte et al., 2006; Kriegeskorte and Bandettini, 2007). We used “The Decoding Toolbox” (Hebart et al., 2015) together with our own custom made code in Matlab to carry out this searchlight analysis. The classification model (SVM) and its parameters for the ROI-based decoding were similar to the ones used for the subject classification analysis.

During the searchlight analysis, a sphere with a radius of two voxels (volume of max. 33 voxels) was sequentially moved across the entire grey-matter volume (Bulthé et al. (2014a). For each participant, the searchlight analysis resulted in a map for each format. Afterwards, the maps were spatially smoothed using Gaussian kernels of 8 mm FWHM (equal to the univariate smoothing level). Finally, a second-level analysis was done in SPM12 to test for group differences and this was done for both formats (threshold of *p* < 0.05 after FWE correction).

##### ROI-based decoding of neural magnitude representations

For each ROI, a decoding classification analysis was implemented with custom code written in Matlab using the LIBSVM toolbox (Chang and Lin, 2011). The classification model (SVM) and its parameters for the ROI-based decoding were similar to the ones used for the subject classification and searchlight analyses.

The response patterns, containing the *t*-values of all voxels for all conditions and each run in that ROI, were obtained. These response patterns were subsequently standardized by subtracting the mean across voxels and then dividing this by the standard deviation across voxels for each condition. We followed a repeated random subsampling cross-validation procedure: The data were randomly divided into 70% training data and 30% test data (the latter were averaged to one response pattern per condition), and this was repeated 100 times.

The pairwise decoding of all eight conditions resulted in an 8 × 8 matrix, for which we then averaged across several decoding accuracies to obtain two comparisons of interest: symbolic numbers (mean within-format decoding accuracy for symbolic numbers) and non-symbolic numbers (mean within-format decoding accuracy for non-symbolic numbers). It is important to note that each time we mention “decoding of non-symbolic numbers” or “decoding of symbolic numbers”, this represents the average across multiple pairwise decoding accuracies. Group differences were tested with a two-sample *t*-test and corrected for multiple comparisons with FDR for the four lobes and for the IPS subparts separately.

#### Functional connectivity analysis

Pre-processing steps for this analysis comprised: (1) bandpass filtering between 0.01 and 0.2 Hz (Balsters et al., 2016; Baria et al., 2013); (2) regression of head motion parameters and their first derivatives; (3) regression of white matter and ventricle signals and their first derivatives (Ebisch et al., 2013); (4) regression of task-related BOLD fluctuations (task = the contrast ‘task minus baseline’) (Boets et al., 2013; Ebisch et al., 2013); (5) scrubbing of motion-affected functional volumes (Power et al., 2012); and (6) spatial smoothing at 4 mm FWHM.

The eight ROIs of level III, which were created for each participant for the analyses described above, were included as seed ROIs for the functional connectivity analysis. We obtained a representative BOLD time course for each ROI by averaging the time courses of the voxels within the ROI. For each participant, we then created a functional connectivity matrix by calculating Pearson cross-correlations between the BOLD time courses of each pair of ROIs. After converting the single-subject matrices to Z-scores with the Fisher's *r*-to-*Z* transformation, we calculated a group-level matrix by conducting a random effects analysis across subjects (*p*_FDR_ < .001). Group-level comparisons among functional connectivity scores were performed by calculating independent-sample *t* tests on the Z-score matrices (*p*_FDR_ < .05).

### DTI data acquisition and analyses

#### DTI data acquisition

DTI data were collected for 23 control participants and 21 participants with dyscalculia. Diffusion images were acquired on a 3T Philips Ingenia CX Scanner using a single spin shot EPI with SENSE acquisition. Whole brain images were acquired with the following parameters: 58 sagittal slices, slice thickness = 2.5 mm, voxel size = 2.5 × 2.5 × 2.5 mm³, repetition time = 7600 ms, echo time = 82 ms, field-of-view = 220 × 240 mm^2^, matrix size = 80 × 94 and acquisition time = 10 min 32 s. Diffusion gradients were applied along 60 noncollinear directions (*b* = 1500 s/mm^2^).

#### Image preprocessing and tractography

Preprocessing of the raw diffusion MRI data was done with ExploreDTI (Leemans et al., 2009) and contained following steps: (1) Images were corrected for eddy current distortion and subject motion; (2) A non-linear least square method was applied for diffusion tensor estimation; (3) For each participant a whole brain tractography was estimated using the following parameters: uniform 2 mm seed point resolution, FA threshold of 0.2, angle threshold of 40°, and fiber length range of 50–500 mm.

We used the TrackVis software to delineate white matter tracts for each participant in their native brain space (Wang and Wedeen, 2007). Based on the review of Matejko and Ansari (2015), the following tracts were delineated: genu and splenium of the corpus callosum (CC), left and right inferior fronto-occipital fasciculus (IFOF), left and right inferior longitudinal fasciculus (ILF), left and right arcuate fasciculus (AF), which was divided into three parts, i.e. frontal to temporoparietal arcuate fasciculus (AF_FTP_), frontal to temporal AF (AF_FT_), frontal to parietal AF (AF_FP_), and left and right temporal to parietal AF (AF_TP_). We opted for deterministic tractography as it is the preferred method for hypothesis-driven tractography. To delineate the tracks, we manually drew ROIs for each individual in their native brain space. These ROIs were defined based on anatomical landmarks in the brain. The delineation of all the tracts for each participant of the control group were done by two independent raters. The inter-rater reliability for all tracts ranged from 0.87 to 0.99, demonstrating a high reproducibility of the tractography.

For all tracts, the fractional anisotropy (FA), mean diffusivity (MD), axial diffusivity (AD), and radial diffusivity (RD) values were extracted for each participant. To test for differences in white matter between the two groups, two sample *t-*tests were performed in Matlab for each DTI metric (FA, MD, AD, and RD values) of each tract. An FDR correction for multiple comparisons was applied across tracts.

## Results

### Group matching

Behavioral and neuroimaging data were collected in 24 individuals with dyscalculia and 24 healthy controls. All adults with dyscalculia met the DSM-V (American Psychiatric Association, 2013) criteria for specific learning disorder with an impairment in mathematics. None of the control participants met the criteria for specific learning disorder. The groups were matched on sex, intelligence, age, educational history and reading ability. As expected, the groups differed significantly in their mathematical abilities (Table 1).

Table 1 presents a summary of the results of all tasks. Two-sample *t*-tests showed that the groups only differed in their performance on the tests measuring mathematical abilities. The results of all these behavioral tests demonstrated an appropriate matching between the two groups.

### Behavioral analysis

A 2 × 2 ANOVA (group x format) was performed to test group and format differences on the number comparison task that was executed during the experimental runs. For accuracy, there was no significant difference between the control group (95.75%, *SD* = 1.97) and dyscalculia group (94.77%, *SD* = 1.39) (*F*_1,92_ = 2.29, p = 0.13, *d*_Cohen_ = 0.45). A significant effect for format was observed and accuracy was higher for symbolic (*M* = 97.35%, *SD* = 1.45) than for non-symbolic numbers (*M* = 93.17%, *SD* = 1.41; *F*_1,92_ = 41.04, p < 0.001, *d*_Cohen_ = 1.89). There was no significant interaction between group and format (*F*_1,92_ = 0.01, *p* = 0.92, *d*_Cohen_ = 0.03).

For reaction time, a significant group difference was observed, with faster response times for controls (*M* = 0.90s, *SD* = 0.07) than for individuals with dyscalculia (*M* = 1.18s, *SD* = 0.07) (*F*_1,92_ = 41.2, *p* < 0.001, *d*_Cohen_ = 1.89). Again, a significant format effect was observed with faster reaction times for symbolic numbers (0.91s, *SD* = 0.05) than for non-symbolic numbers (1.17s, *SD* = 0.08) (*F*_1,92_ = 34.67, *p* < 0.001, *d*_Cohen_ = 1.74). There was no interaction between group and format (*F*_1,92_ = 0.36, *p* = 0.55, *d*_Cohen_ = 0.17).

The two groups performed the task equally well in terms of accuracy, but individuals with dyscalculia were significantly slower compared to the control group. It should be noted that a similar behavioral pattern was observed in a multi-method imaging study on impairments in dyslexia (Boets et al., 2013).

### Neural activation levels for symbolic and non-symbolic numbers

#### Univariate analyses

As a first step, and similar to earlier studies, we performed univariate analyses to investigate group differences in the overall activation level. No significant group differences for symbolic and non-symbolic numbers were found with a whole brain voxel-wise *t*-test (second-level analysis, voxel-wise FWE corrected at *p* < 0.05, height threshold *T* = 5.29, extent threshold *k* = 0 voxels).

For the ROI-based univariate analysis, we found no significant group differences in brain activity for symbolic and non-symbolic formats in the All Regions analysis (Dots: *t*_46_ = 1.44, *p* = 0.11; Arabic digits: *t*_46_ = 1.86, *p* = 0.07). There were also no significant differences in activity in any of the individual ROIs (lowest *p*_*FDR*_ = 0.10).

#### Subject classification

To further investigate whether there were distinguishable patterns of activation versus fixation between the two groups, we applied a subject classification procedure (see Methods). Subject classification allowed us to examine if the activation patterns of both groups for either formats at various spatial levels were different enough to be picked up by a classifier.

The results of the subject classification analysis did not show a significant subject classification accuracy, with the same patterns in the All Regions ROI, for both non-symbolic (classification accuracy = 0.57, *p* = 0.17) or symbolic (classification accuracy = 0.51, *p* = 0.45) numbers. None of the ROIs showed a significant effect at an FDR-corrected level for both formats. Even with these sensitive classification methods, we did not find significant differences between the participant groups in terms of their general pattern of activation. These analyses suggest that the same representations and processes were involved in the two groups.

#### Summary of univariate results

We found no univariate differences in brain activity between adults with and without dyscalculia. A whole-brain second level univariate analysis did not reveal any significant activity level differences between the groups. A subsequent ROI-based analysis in 17 ROIs confirmed the whole-brain univariate finding of no group differences. We also applied a subject classification analysis to verify whether there were more distributed and subtle activation differences between both groups. Again, this analysis did not reveal significant group differences. Taking these three analyses together, we conclude that there were no significant univariate differences between adults with and without dyscalculia during number processing.

### Quality of neural representations of symbolic and non-symbolic numbers

#### Searchlight analysis

The abovementioned results revealed no significant group differences in the level of activation for either numerical format. Previous research that compared the brain activity of individuals with and without dyscalculia (Ashkenazi et al., 2012; Mussolin et al., 2010b; Price et al., 2007; Rosenberg-Lee et al., 2015; Simos et al., 2008) only considered univariate comparisons. In this section, we conducted more refined analyses that allowed us to assess the quality of neural representations. To this end, we applied a whole-brain MVPA searchlight analysis based upon the decoding of different magnitudes. This analysis was performed separately for the non-symbolic and symbolic magnitude representations.

**Fig. 3:**
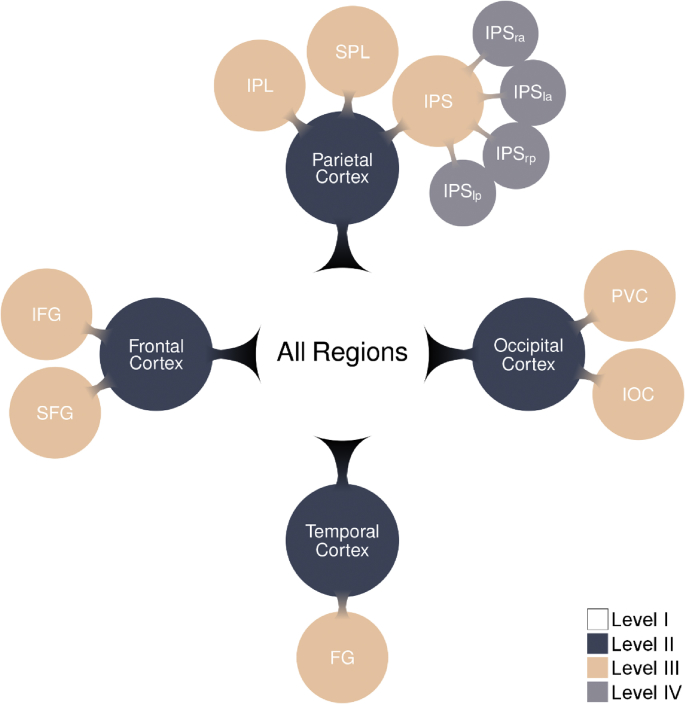
Overview of the hierarchical structure for the ROIs. ROIs that belonged in the same level are indicated by the same color. Only in the case of a significant group effect at the higher level, the lower level was tested for both groups. Each time, FDR correction was applied for all the ROIs belonging to the same level. Abbreviations: left anterior IPS (IPS_la_), right anterior IPS (IPS_ra_), left posterior IPS (IPS_lp_), and right posterior IPS (IPS_rp_).

The results of these searchlight analysis showed specific ‘hotspots’ in both groups for non-symbolic and symbolic number representations. In controls, the non-symbolic representations of various numerosities (e.g., 4 dots versus 8 dots) were distinguishable in many regions of the dorsal stream (Fig. 3A). In individuals with dyscalculia, the non-symbolic number representations were distinct in the occipital pole and in a few patches in the parietal cortex (mainly superior parietal lobule) (Fig. 3B).

A group comparison for non-symbolic numbers (Fig. 3C–D) clearly demonstrated significantly less distinct non-symbolic number representations in the anterior parietal and frontal lobes, as well as in a small spot in the temporal lobe in individuals with dyscalculia compared to controls.

For symbolic numbers, we found fewer regions with distinct number representations for both groups, compared with non-symbolic numbers (Fig. 3C–D). This is consistent with earlier studies using a similar paradigm and data-analytic methods (Eger et al., 2009; Damarla and Just, 2013; Bulthé et al., 2014a, Bulthé et al., 2014b), which also showed a much lower ability to decode symbolic numbers compared to non-symbolic numbers. The searchlight maps of both groups were not significantly different for symbolic numbers. In other words, there appears to be no group difference in the overlap of the neural representations.

In both groups, the classifier was also able to distinguish between symbolic numbers in the occipital pole. This is likely due to the lack of being able to fully control for extensive and intensive visual features in these stimuli. The classifier was also able to distinguish between symbolic numbers in the motor and somatosensory cortex, but this significant accuracy might be due to a response confound. Most (four out of six) pairwise comparisons of numbers were between conditions that triggered different motor responses (e.g. smaller or larger than 5) and this might have been picked up by the classifier. Note that in a different study with the same paradigm and only 16 subjects, we did not observe this effect (Bulthé et al., 2014a). This suggests that it was a small effect that can only be picked up by the classifier when there is a sufficient sample size. No group differences were observed in any of these regions.

#### ROI-based decoding

The searchlight results should not be interpreted as evidence that number representations are relatively focal, because these searchlight analyses are notoriously biased towards finding focal representations (Bulthé et al., 2014b). We therefore applied ROI-based decoding analyses to look for more widespread differences between the two groups. This was first done on larger spatial scale (All Regions and different lobes) and subsequently on smaller spatial scales (IPS, temporal, frontal, and occipital regions).

To test whether there were distinct underlying neural representations for symbolic and non-symbolic numbers in each ROI, a classifier was trained and tested to differentiate between the different numerical magnitudes in one format in a given ROI. If there were distinct neural representations in that ROI, the decoding accuracies should be significant, with higher decoding accuracies indicating more distinct neural representations. We also compared the decoding accuracies of both groups for each format.

##### All regions

**Fig. 4:**
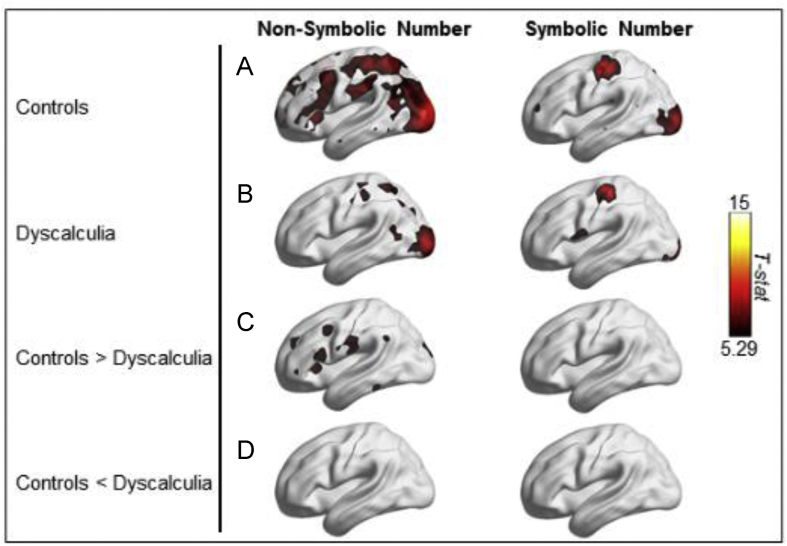
Searchlight results. Decoding accuracies elicited by non-symbolic and symbolic numbers in the both groups. The difference in decoding accuracies between the two groups for each format is shown. The results were corrected with FWE (p < 0.05), height threshold T = 5.29, extent threshold k = 0 voxels. An overview of the peak voxels of these findings is included in the supplementary material (Table S1).

The classifier could distinguish the neural representations of non-symbolic and symbolic numbers in both groups (both *p*-values < 0.001) (Fig. 4 & Table S2-S3). For non-symbolic numbers, the neural representations were more separable in controls than in dyscalculia (*t*_46_ = 2.16, *p* = 0.04). For symbolic numbers there was no significant difference in the quality of the neural representations between controls and dyscalculia (*t*_46_ = −0.56, *p* = 0.58). Because decoding performance was markedly lower for symbolic than for non-symbolic numbers, with decoding performance going down from 63% to 47% overall, this absence of a significant group difference for symbolic numbers might have been due to a lack of sensitivity to detect a possible group difference.

##### Lobes

The decoding accuracies for non-symbolic numbers were significantly different from chance level in both groups in all lobes (all *p*_FDR_'s < 0.001) (Fig. 4 & Table S2-S3). There was no significant group difference in the occipital lobe (*p*_*FDR*_ = 0.11). A significant group difference was observed in the parietal lobe, temporal lobe, and frontal lobe (all *p*_FDR_'s < 0.03), with more distinguishable neural patterns for non-symbolic numbers in controls than in individuals with dyscalculia.

##### Parietal ROIs

The neural patterns for non-symbolic numbers were distinct from each other in each group in the IPL, SPL, and the IPS (all *p*_FDR_'s < 0.001) (Fig. 4 & Table S2-S3). Although there was a strong trend for more distinguishable neural patterns in controls than in individuals with dyscalculia in the IPL (*p*_*FDR*_ = 0.052) and SPL (*p*_*FDR*_ = 0.052), the group difference, with more distinct neural patterns in controls than in dyscalculia, was only significant in the IPS (*p*_*FDR*_ = 0.048).

##### IPS subparts

Because there was a significant group difference for non-symbolic numbers in the IPS (Fig. 4 & Table S2-S3), its subparts were also analyzed for presence of distinct non-symbolic number representations in each group. We also tested whether there was a group difference in the distinctiveness of these neural representations. In all the subparts of the IPS the classifier reliably differentiated between the different neural representations of non-symbolic numbers in each group (all *p*_FDR_'s < 0.001) (Fig. 4 & Table S2-S3). Only in the IPS_RA_, the distinctiveness of the neural representations was significantly higher for controls than for individuals with dyscalculia (*p*_*FDR*_ = 0.02).

##### Temporal ROIs

The FG contained distinct neural representations for non-symbolic numbers in both groups (both *p*_FDR_'s < 0.001) (Fig. 4 & Table S2-S3). There was no group difference in the distinctiveness of these neural representations (*p*_*FDR*_ = 0.55).

##### Frontal ROIs

The classifier distinguished different neural representations for non-symbolic numbers for both groups in the IFG and the SFG (both *p*_FDR_'s < 0.001) (Fig. 4 & Table S2-S3). The neural representations in the IFG and the SFG in controls were also more distinct than in individuals with dyscalculia (both *p*_FDR_'s < 0.03).

#### Summary of multivariate results

We first applied a searchlight analysis, which investigated the processing of symbolic and non-symbolic numbers on a very local scale. These analyses demonstrated less distinguishable non-symbolic number representations in adults with dyscalculia compared to adults without dyscalculia, in the anterior parietal lobe, the frontal lobe, and in a small area in the temporal lobe. For symbolic number representations, we did not observe any group differences in the quality of the representations.

The searchlight results were confirmed by the ROI-based multivariate approach. For non-symbolic numbers, we found significantly less distinguishable neural representations in adults with dyscalculia compared with matched controls in the All Regions, the parietal lobe (including the IPS and right anterior IPS), the temporal lobe, and the frontal lobe (including superior and inferior frontal gyrus). For symbolic numbers, we did not observe any significant group differences.

### Connectivity differences between the two groups?

We subsequently tested if the numerical impairments in dyscalculia were due to an access deficit and, consequently, to differences in connectivity. This was done by analyzing (A) functional connectivity between ROIs and (B) structural connectivity in specific white matter tracts (Fig. 1c).

#### Functional connectivity

**Fig. 5:**
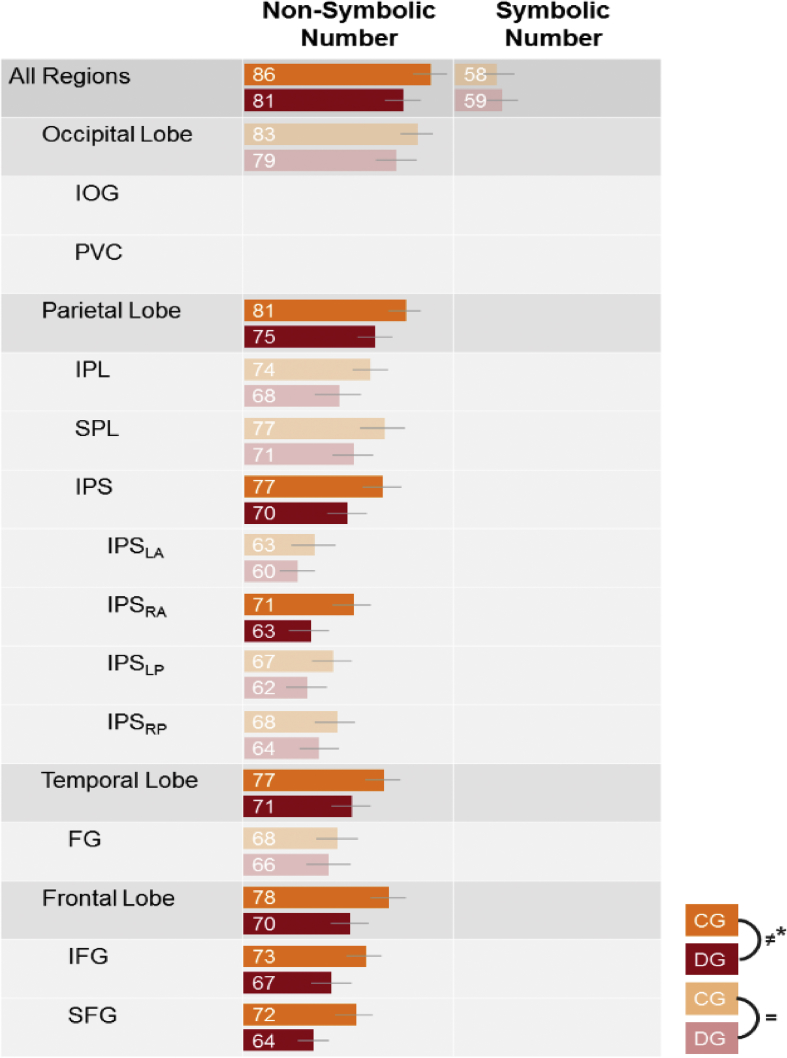
Decoding accuracies of controls and individuals with dyscalculia for non-symbolic and symbolic numbers for each ROI, in hierarchical order. Orange represents the control group (CG) and red denotes the group with dyscalculia (DG). All the within-group decoding accuracies were significantly different from chance level (FDR corrected). The dark colored bars show a significant group difference between controls (orange) and dyscalculia (red) (FDR corrected). If a higher order ROI did not show a significant group difference (dimmed bars), lower ROIs were not analyzed and they are not shown in the figure. Error bars represent the 95% confidence interval for the decoding accuracy for that group in that ROI.

A functional connectivity analysis was performed to investigate the ROIs that were functionally coupled with each other and to test whether this coupling differed between the two groups. Only the 8 ROIs on level III (Fig. 2) were included. For both groups, all the pairwise functional connectivity strengths were significant (controls: 10.92 < *t*_23_ < 38.81, all *p*_FDR_'s < 0.001; dyscalculia: 12.80 < *t*_23_ < 37.58, all *p*_FDR_'s < 0.001) (Fig. 5a–b). For the individual connections between ROIs, there were significant group differences for the connectivity between PVC (primary visual cortex) and IOC (t46 = −3.17, pFDR = 0.04) and between PVC and FG (t46 = −3.47, pFDR = 0.03) with higher connectivity in individuals with dyscalculia than in controls (Fig. 5c). Interestingly, none of the regions involved in this hyper-connectivity showed a group difference in the distinctiveness of neural representations in the previous analyses.

**Fig. 6:**
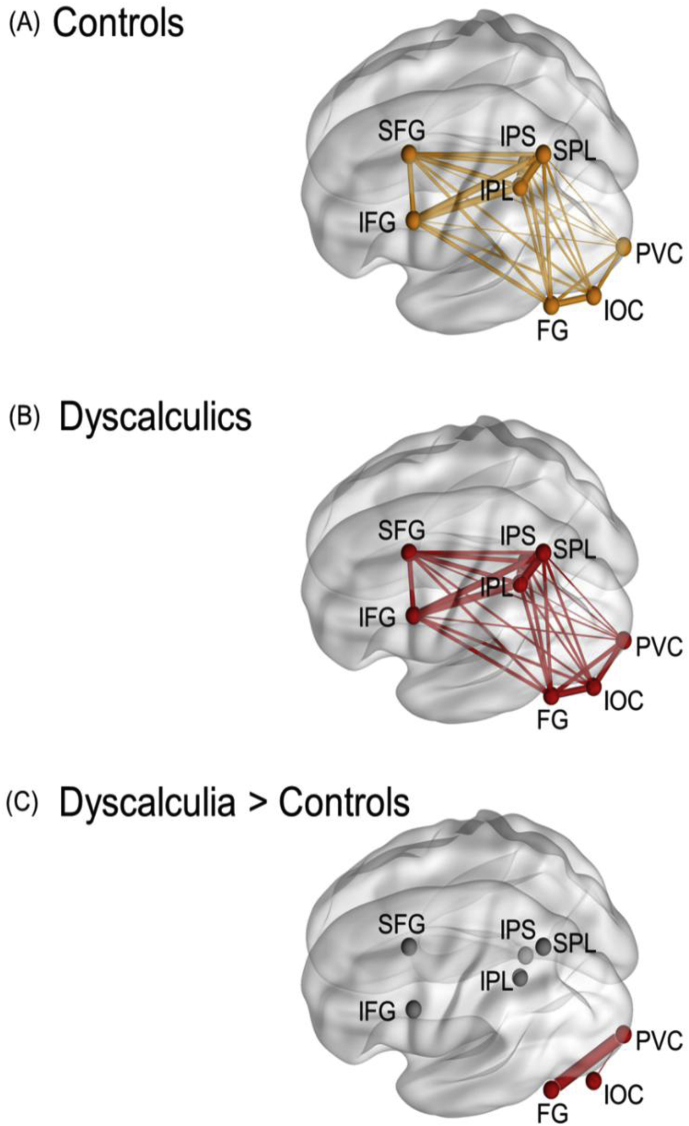
Overview of the fcMRI results. All the ROIs were significantly functionally connected with each other for (*A*) control participants and (*B*) participants with dyscalculia at an FDR (*p* < 0.001) corrected level. (*C*) We observed a significant group difference at FDR (*p* < 0.05) corrected level, with higher functional connectivity for dyscalculia between PVC and IOC and between PVC and FG. The thickness of the lines represents the strength of the connections.

#### Structural connectivity

DTI measures the quality of the white matter tracts that connect different regions in the human cortex. None of the tracts under investigation showed a significant group difference (even at uncorrected level for multiple comparisons) for fractional anisotropy (FA), mean diffusivity (MD), axial diffusivity (AD), and radial diffusivity (RD) values. For FA, the *t-*values ranged from −1.85 to 1.41 (0.41 < *p*_*FDR*_ < 0.91) (see supplementary material, Figure S1); for AD *t*-values ranged from −1.85 to 0.82 (0.54 < *p*_*FDR*_ < 0.99); for MD *t-*values ranged from −0.92 to 0.90 (all *p*_*FDR*_'s = 0.97); and for RD *t*-values ranged from −0.79 to 1.39 (0.89 < *p*_*FDR*_ < 0.98).

#### Summary of connectivity results

The results of the functional connectivity analysis showed an increased connectivity between the primary visual cortex and fusiform gyrus, and between primary visual cortex and inferior occipital complex for adults with dyscalculia compared to controls. For the structural connectivity analysis, we did not observe any significant group differences.

## Discussion

This study combined for the first time different neuroimaging methods (univariate and multivariate functional, and connectivity analyses) to investigate the impaired number representations hypothesis and the impaired access to number representations hypothesis in relation to dyscalculia. Our results indicate impaired non-symbolic magnitude representations in the IPS, parietal regions, and other brain regions in adults with dyscalculia. This is consistent with the impaired magnitude representations hypothesis. We also found functional connectivity differences in adults with dyscalculia, in line with the hypothesis of impaired access to number representations. These differences in connectivity were observed in brain regions that did not show a difference in the quality of magnitude representations. The neural correlates of dyscalculia in adults cannot be reduced to only impaired magnitude representations or to only impaired access to these magnitude representations. Our data further indicate that they also cannot be reduced to one or a few brain areas.

The observation that the deficits in dyscalculia cannot be localized to one brain region or cannot be explained by a single brain deficit has important consequences for the study of the neural basis of dyscalculia. This is especially so, because previous studies have either focused narrowly on the IPS and parietal regions or on one specific type of analyses, such as univariate analyses. Our findings are consistent with the idea that dyscalculia, just like other neurodevelopmental disorders, such as dyslexia, ADHD or autism (Pennington, 2006), is a heterogeneous disorder that arises from multiple risk and protective factors. As a consequence, its neural correlates will include combinations of diffuse functional disruptions, deficits in connectivity between regions, as well as anatomical differences (see also Johnson et al., 2002; Menon, 2011).

### Functional impairments

We demonstrated that the deficits in the quality of neural magnitude representations are spread throughout the cortex, as was already suggested on the basis of more indirect evidence from univariate studies (Kaufmann et al., 2011; Rosenberg-Lee et al., 2015). In the current study, the representations in these regions outside the parietal cortex might be related to numerical representations as well as to how these representations are recruited during more general cognitive processes. For example, frontal regions often play an important role in attention and working memory processes required for problem solving, and these processes are also likely to occur when dealing with numerical magnitudes.

We demonstrated impaired quality of non-symbolic magnitude representations while participants were performing a numerical magnitude comparison task. We deliberately opted for timing parameters that allowed participants with dyscalculia to perform the task quite well. Their overall accuracy was not significantly different from controls, with a difference only observed for reaction times. Because the overall accuracy in individuals with dyscalculia was not different from matched controls, the impaired quality of magnitude representations at the neural level cannot be explained by one group not being able to perform the task and hence being less motivated/attentive. The slower reaction time of the participants with dyscalculia suggests that they were processing the stimuli for a longer period of time, which could have resulted in a better quality of the magnitude representations – the opposite of what we observed. Interestingly, a previous multi-method imaging study of phonological processing in dyslexia also compared participant groups with the same accuracy but different reaction time (slower in individuals with the dyslexia) (Boets et al., 2013). In that study there were no group differences in the quality of neural representations between individuals with and without dyslexia.

Although the deficit in non-symbolic number processing for dyscalculia was very clear in our study, we observed no group differences in the quality of magnitude representations for symbolic numbers. This does not necessarily mean that symbolic representations are not impaired in dyscalculia. Previous MVPA studies in healthy participants already showed that these symbolic representations are harder to detect than representations of non-symbolic magnitudes (Eger et al., 2009; Damarla and Just, 2012; Bulthé et al., 2014a, 2015; Lyons et al., 2015). This was also observed in the current study as we found smaller decoding accuracies, also in controls, for symbolic magnitudes. Because decoding performance was markedly lower for symbolic than for non-symbolic numbers, the lack of a significant group difference for symbolic numbers might be explained by a lack of sensitivity to detect a possible group difference. Future studies on the quality of symbolic number representations in dyscalculia should therefore collect more neuroimaging data of symbolic numbers. Another possibility to increase sensitivity of the imaging design might be to increase the difficulty of the task, for example by using two-digit symbolic numbers.

Our study differs from previous neuroimaging studies on dyscalculia in two respects. First, previous neuroimaging studies used univariate fMRI analyses to investigate differences in brain activation between individuals with dyscalculia and matched controls. Applying the same type of univariate analyses to our data as in previous studies, we failed to detect any clusters of voxels in the brain that showed a different level of activation in individuals with dyscalculia vs. healthy controls. It is not clear whether and in what direction our univariate null results would deviate from earlier observed task-dependent altered levels of activations in dyscalculia as mixed results have been reported. Several studies have reported a decreased activation in dyscalculia during non-symbolic number comparison tasks (Price et al., 2007), arithmetic problem solving (Ashkenazi et al., 2012), and symbolic number comparison (Mussolin et al., 2010a). On the other hand, Rosenberg-Lee et al. (2015) have reported increased activity in the IPS during arithmetic problem solving in children with dyscalculia compared to matched controls. Iuculano et al. (2014) found hyperactivity in children with dyscalculia compared to matched controls in multiple cortical areas in the prefrontal and parietal cortex (including the left IPS), and ventral temporal-occipital cortex. These mixed findings might be due to differences in task domains, task difficulty or samples. Therefore, no obvious and unequivocal prediction can be made about the direction of univariate differences in the neural response of individuals with dyscalculia when comparing them to healthy controls.

Second, all previous functional neuroimaging work on dyscalculia has been carried out with children. The current study is the first neuroimaging study with adults with dyscalculia. Hence, it was not known to date whether this altered functional activation levels in the IPS and other brain regions associated with dyscalculia remain altered throughout the lifespan. Our results suggest that such differences in overall activity may have diminished during adulthood. This does not exclude the possibility that during development there were time points during which hyper- or hypoactivation in the IPS in children with dyscalculia could have been observed. Related to this issue, Iuculano and colleagues (2015) have demonstrated that the difference in brain activity between children with and without mathematical learning disabilities diminished after remedial interventions. This suggests that even in dyscalculia, brain activity can normalize after successful remediation, a possibility that also has been observed in dyslexia (Temple et al., 2003). On the other hand, group differences in overall brain activity between adults with and without dyscalculia might only emerge when more complex numerical tasks, such as calculation rather than number comparison are used.

To conclude, our multivariate analyses results provided the first evidence that non-symbolic number representations were less precise in adults with dyscalculia in comparison to a strictly matched control group. Our findings of impaired number representations in adults with dyscalculia are observed at both the distributed and the local scale. The ROI-based MVPA clearly demonstrated less precise non-symbolic number representations in dyscalculia throughout the dorsal stream and the IPS, and therefore showed a very distributed neural correlation with number representations in dyscalculia. On the other hand, the searchlight analysis demonstrated several local areas in the temporal, parietal and frontal cortex, where less precise non-symbolic number representations were present in adults with dyscalculia compared to adults without dyscalculia. For symbolic magnitudes we did not observe differences in neural quality between the two groups under study. Further investigation is needed to determine whether the symbolic neural representations are less precise in dyscalculia.

### Connectivity deficits

The current study is the first neuroimaging study to consider the structural and functional connectivity correlates in adults with dyscalculia. We observed increased functional connectivity in temporal/occipital regions in adults with dyscalculia. However, we found no group differences in any of the white matter tracts (four in total with 17 segments) under investigation.

We observed hyper-connectivity in adults with dyscalculia, which is in line with functional connectivity (Rosenberg-Lee et al., 2015) and resting-state connectivity (Jolles et al., 2016) data in children with dyscalculia. The hyper-connectivity in our study was observed between FG and PVC and between IOC and PVC, regions known to be involved in the processing of complex visual objects (Grill-Spector et al., 2008; Menon et al., 2000). It has been previously demonstrated that children with dyscalculia have decreased grey matter volume in these regions (Rykhlevskaia et al., 2009). The observed increased connectivity in the study of Rykhlevskaia et al. (2009) was related to arithmetic skills, such that more functional connectivity in these regions was associated with lower arithmetic skills.

The increased functional connectivity in dyscalculia in our study might be interpreted in terms of compensatory processes that were activated during digit number processing in dyscalculia. More specifically, this increased functional connectivity from the occipital cortex to the inferior-temporal cortex might be explained by increased connectivity to the “visual number area” (located in the inferior-temporal cortex). This area was first identified by Shum et al. (2013) who collected intracranial electrophysiological recordings. Shum et al. (2013) demonstrated that this area responds more strongly to digits than to other stimuli, which were well-matched in terms of visual (letters, false fonts), semantic (number words) or phonological (phonologically similar non-number words) similarity. Using monkey fMRI, Srihasam et al. (2012) also found an area in the ventral temporal cortex selective for trained symbols (opposed to untrained shapes and faces). These results suggest that such regions only develop when a high level of visual proficiency with number symbols is reached (as was the case for the younger monkeys in the study of Shrihasam et al. (2012). Against this background, we hypothesize that the observed increased connectivity in the direction of the inferior-temporal cortex might reflect compensatory mechanisms in individuals with dyscalculia to process Arabic digits. It is important to emphasize that we did not study the exact location of this “visual number area”, as this region is close to the fMRI signal-drop out zone produced by the nearby auditory canal and venous sinus (Shum et al., 2013). On the other hand, recent data by Grotheer et al. (2018) suggest that it is possible to investigate brain activity in this inferior temporal area via fMRI and that this region may be involved in mathematical processing more broadly. Future studies should therefore more carefully investigate this region and examine the possibility of compensatory mechanisms is this region in dyscalculia.

In contrast to the functional connectivity differences in this study, we did not observe any differences in structural connectivity in tracts that were previously related to numerical processing (Matejko and Ansari, 2014). This is in contrast to two previous studies in dyscalculia, who observed that children with dyscalculia had decreased connectivity in the right temporal-parietal areas (Rykhlevskaia et al., 2009), and the bilateral superior longitudinal fasciculus (Kucian et al., 2013). There are two possible explanations for the discrepancy in findings. First, it might be that the white matter differences observed in children with dyscalculia are only present early in development, and that over time these white matter differences become very small or even negligible. Second, the present study included a very strictly matched control group that took into account not only sex, age, and intelligence, but also educational history and environment. This strict matching might also explain why we did not replicate previous neuroimaging studies in which such matching was not employed. However, this strict matching is critical to attribute the observed differences to the presence of dyscalculia and not to possible differences in education.

### Domain-specific vs. domain-general deficits

There is an increasing awareness that the neural origins of dyscalculia may not be restricted to domain-specific deficits, such as impaired representations of number or deficits in access to these domain-specific representations. In addition, the neural correlates of dyscalculia might also include domain-general deficits and impairments that are not specific to learning mathematics (Ashkenazi et al., 2013; Fias et al., 2013; Geary et al., 2017; Rubinsten and Henik, 2009; Szűcs, 2013; Wilson et al., 2015) For example, impairments in executive functions, working memory, attention or inhibitory control all have been highlighted as potential risk factors for dyscalculia (Rubinsten and Henik, 2009).

Our multi-faceted analyses might point to both domain-specific and domain-general deficits. Impaired neural representations for non-symbolic magnitudes in adults with dyscalculia in brain regions that are typically correlated with numerical cognition (IPS and parietal cortex), might point to domain-specific impairments. On the other hand, various observations suggest domain-general effects. For example, the impaired neural magnitude representations in dyscalculia were not *only* located in the IPS and parietal regions. As in previous studies, we demonstrated that deficits in functional neural correlates of number in dyscalculia were spread out throughout the cortex (Kaufmann et al., 2011; Rosenberg-Lee et al., 2015; Rotzer et al., 2008; Rykhlevskaia et al., 2009). In our study, the representations in (some of) these extra-parietal regions might be related to numerical representations as well as to how these representations are recruited during more domain-general processes. For example, frontal regions often play an important role in attention and working memory processes required for problem solving, and this also applies to processing information in a numerical context. Our functional connectivity results showed hyper-connectivity in dyscalculia between visual regions and fusiform gyrus. Interestingly, increased functional connectivity was previously linked to compensation processes and inhibitory processes (Geerligs et al., 2012; Rosenberg-Lee et al., 2015). It is important to acknowledge that these interpretations should be made with caution, as they might be reflective of reverse inference, and future studies are needed to directly test these interpretations.

### Limitations

Some limitations should be kept in mind when evaluating the results of the current study. The behavioral data showed trends towards group differences in reading, IQ and motor reaction time, yet all of these effects were small. It is, however, important to emphasize that all scores on these measures were in the normal range and that none of the participants with dyscalculia had impairments in these abilities. These small trends could not explain the individual impairments in mathematics in the participants with dyscalculia.

Our data only included female participants. We did not initially intend to exclude male adults from our study. Our public call for participants was directed to all individuals with dyscalculia, independent of sex. We would like to point out that there have been many other studies on dyscalculia with only or mainly female participants (all female: Defever et al., 2014 and Vanbinst and De Smedt, 2016; one male: Cappelletti et al., 2011; mainly females: Mussolin et al., 2011). To the best of our knowledge, epidemiological studies do not indicate a strong gender bias towards females in dyscalculia (for a review, see Devine et al., 2013), although it needs to be noted that girls are more diagnosed with dyscalculia than boys (Moll et al., 2014).

We reported the overall functional connectivity across all task conditions. Future studies should investigate functional connectivity in much more detail by examining it in relation to specific task manipulations and by comparing it to pure (i.e. task-independent) resting-state connectivity as acquired by additional resting state fMRI. First, it is relevant to search for task-dependent differences in functional connectivity and their association with dyscalculia. This would require, however, a different analytic approach than in the current study. We analyzed our data with the typical approach to analyze functional connectivity in resting-state data (after regressing out the univariate task activations). The extension of this approach to study interactions with the task requires that the different tasks are presented in blocks of a long duration, which was not the case in the present paradigm. An alternative is the psycho-physiological interaction approach that mainly tests for fluctuations in activity across trials modeled in a regression approach. Second, there are also potential interactions between magnitude representations and functional connectivity that can be discovered by the so-called multivariate connectivity analyses that were introduced recently (Anzellotti and Coutanche, 2018). Given that we observe that dyscalculia is characterized by less distinctive magnitude representations, it is not unlikely that the temporal fluctuations in these magnitude representations will also be affected. Future studies are needed to further test these predictions.

### Conclusion

Our study supports a clear deficit in the quality of non-symbolic magnitude representations in adults with dyscalculia, confirming the impaired number representations hypothesis. We also observed hyper-connectivity in visual brain regions in adults with dyscalculia, which is in line with the impaired access hypothesis. The deficits in dyscalculia were not be localized in one particular brain region (such as the IPS). They could not be reduced to one type of brain deficit (such as only impaired number representations). Our study illustrates the many advantages of combining different imaging techniques at the whole brain level to disentagle the neural correlates of neurodevelopmental disorders compared to studies with a focus on only one brain region and/or only one neuroimaging method. Future studies should therefore continue to apply multi-method approaches to investigate neurodevelopmental disorders and mental disorders at large, in adults as well as children.

## Appendix A Supplementary data

Bulthe_supplementary_material_V2 Bulthe_supplementary_material_V2

## References

[bib1] American Psychiatric Association (2013). Diagnostic and statistical manual of mental disorders. American Psychiatric Association.

[bib2] Anzellotti S., Coutanche M.N. (2018). Beyond functional connectivity: investigating networks of multivariate representations. Trends Cogn. Sci.

[bib3] Ashkenazi S., Rosenberg-Lee M., Tenison C., Menon V. (2012). Weak task-related modulation and stimulus representations during arithmetic problem solving in children with developmental dyscalculia. Dev. Cogn. Neurosci.

[bib4] Balsters J.H., Mantini D., Apps M.A.J., Eickhoff S.B., Wenderoth N. (2016). Connectivity-based parcellation increases network detection sensitivity in resting state fMRI: an investigation into the cingulate cortex in autism. NeuroImage Clin.

[bib5] Baria A.T., Mansour A., Huang L., Baliki M.N., Cecchi G.A., Mesulam M.M., Apkarian A.V. (2013). Linking human brain local activity fluctuations to structural and functional network architectures. Neuroimage.

[bib6] Bishop D.V.M. (2010). Which neurodevelopmental disorders get researched and why?. PLoS One.

[bib7] Boets B., Op de Beeck H.P., Vandermosten M., Scott S.K., Gillebert C.R., Mantini D., Bulthe J., Sunaert S., Wouters J., Ghesquiere P. (2013). Intact but less accessible phonetic representations in adults with dyslexia. Science (Wash. D C).

[bib8] Brainard D.H. (1997). The psychophysics toolbox. Spat. Vis.

[bib9] Brus B. (1999). Een-minuut-test.

[bib10] Bulthé J., De Smedt B., Op de Beeck H.P. (2015). Visual number beats abstract numerical magnitude: format-dependent representation of Arabic digits and dot patterns in human parietal cortex. J. Cognit. Neurosci..

[bib11] Bulthé J., De Smedt B., Op de Beeck H.P. (2014). Format-dependent representations of symbolic and non-symbolic numbers in the human cortex as revealed by multi-voxel pattern analyses. Neuroimage.

[bib12] Bulthé J., van den Hurk J., Daniels N., De Smedt B., Op de Beeck H.P. (2014). A validation of a multi-spatialscale method for multivariate pattern analysis. 2014 International Workshop on Pattern Recognition in Neuroimaging.

[bib13] Burr D., Ross J. (2008). A visual sense of number. Curr. Biol..

[bib14] Butterworth B., Varma S., Laurillard D. (2011). Dyscalculia: from brain to education. Science (Wash. D C).

[bib15] Cappelletti M., Freeman E., Butterworth B. (2011). Time processing in dyscalculia. Front. Psychol..

[bib16] Chang C.-C., Lin C.-J. (2011). {LIBSVM}: a library for support vector machines. ACM Trans. Intell. Syst. Technol. 2.

[bib18] Damarla S.R., Just M.A. (2013). Decoding the representation of numerical values from brain activation patterns. Hum. Brain Mapp..

[bib19] De Smedt B., Boets B. (2010). Phonological processing and arithmetic fact retrieval: evidence from developmental dyslexia. Neuropsychologia.

[bib20] De Smedt B., Gilmore C.K. (2011). Defective number module or impaired access? Numerical magnitude processing in first graders with mathematical difficulties. J. Exp. Child Psychol..

[bib21] De Smedt B., Noël M.-P., Gilmore C., Ansari D. (2013). How do symbolic and non-symbolic numerical magnitude processing skills relate to individual differences in children's mathematical skills? A review of evidence from brain and behavior. Trends Neurosci. Educ.

[bib22] De Vos T. (1992). Tempo-test-rekenen. Handleiding [Tempo Test Arithmetic. Manual]. Nijmegen: Berkhout.

[bib23] Defever E., Göbel S.M., Ghesquière P., Reynvoet B. (2014). Automatic number priming effects in adults with and without mathematical learning disabilities. Front. Psychol..

[bib24] Dehaene S., Izard V., Piazza M. (2005). Control Over Non-Numerical Parameters in Numerosity Experiments. http://www.unicog.org.

[bib25] Devine A., Soltész F., Nobes A., Goswami U., Szűcs D. (2013). Gender differences in developmental dyscalculia depend on diagnostic criteria. Learn. InStruct..

[bib26] Ebisch S.J.H., Mantini D., Romanelli R., Tommasi M., Perrucci M.G., Romani G.L., Colom R., Saggino A. (2013). Long-range functional interactions of anterior insula and medial frontal cortex are differently modulated by visuospatial and inductive reasoning tasks. Neuroimage.

[bib27] Eger E., Michel V., Thirion B., Amadon A., Dehaene S., Kleinschmidt A. (2009). Deciphering cortical number coding from human brain activity patterns. Curr. Biol..

[bib28] Elsabbagh M., Divan G., Koh Y.-J., Kim Y.S., Kauchali S., Marcín C., Montiel-Nava C., Patel V., Paula C.S., Wang C., Yasamy M.T., Fombonne E. (2012). Global prevalence of autism and other pervasive developmental disorders. Autism Res..

[bib29] Estrada-Mejia C., de Vries M., Zeelenberg M. (2016). Numeracy and wealth. J. Econ. Psychol..

[bib30] Fias W., Menon V., Szucs D. (2013). Multiple components of developmental dyscalculia. Trends Neurosci. Educ. Next.

[bib31] French J., Ekstrom R., Price L. (1963). Manual for Kit of Reference Tests for Cognitive Factors (Revised 1963) (Tech. Rep.).

[bib32] Geary D.C., Nicholas A., Li Y., Sun J. (2017). Developmental change in the influence of domain-general abilities and domain-specific knowledge on mathematics achievement: an eight-year longitudinal study. J. Educ. Psychol..

[bib33] Geerligs L., Saliasi E., Maurits N.M., Lorist M.M. (2012). Compensation through increased functional connectivity: neural correlates of inhibition in old and young. J. Cognit. Neurosci..

[bib34] Gerardi K., Goette L., Meier S. (2013). Numerical ability predicts mortgage default. Proc. Natl. Acad. Sci. Unit. States Am..

[bib35] Grill-Spector K., Golarai G., Gabrieli J. (2008). Developmental neuroimaging of the human ventral visual cortex. Trends Cognit. Sci..

[bib36] Grotheer M., Jeska B., Grill-Spector K. (2018). A preference for mathematical processing outweighs the selectivity for Arabic numbers in the inferior temporal gyrus. Neuroimage.

[bib37] Hebart M.N., Görgen K., Haynes J.-D., Dubois J. (2015). The Decoding Toolbox (TDT): a versatile software package for multivariate analyses of functional imaging data. Front. Neuroinform.

[bib38] Holloway I.D., Battista C., Vogel S.E., Ansari D. (2013). Semantic and perceptual processing of number symbols: evidence from a cross-linguistic fMRI adaptation study. J. Cognit. Neurosci..

[bib39] Iuculano T., Rosenberg-Lee M., Richardson J., Tenison C., Fuchs L., Supekar K., Menon V. (2015). Cognitive tutoring induces widespread neuroplasticity and remediates brain function in children with mathematical learning disabilities. Nat. Commun..

[bib40] Iuculano T., Rosenberg-Lee M., Supekar K., Lynch C.J., Khouzam A., Phillips J., Uddin L.Q., Menon V. (2014). Brain organization underlying superior mathematical abilities in children with autism. Biol. Psychiatr..

[bib41] Johnson M.H., Halit H., Grice S.J., Karmiloff-Smith A. (2002). Neuroimaging of typical and atypical development: a perspective from multiple levels of analysis. Dev. Psychopathol..

[bib42] Jolles D., Ashkenazi S., Kochalka J., Evans T., Richardson J., Rosenberg-Lee M., Zhao H., Supekar K., Chen T., Menon V. (2016). Parietal hyper-connectivity, aberrant brain organization, and circuit-based biomarkers in children with mathematical disabilities. Dev. Sci..

[bib43] Kaufmann L., Wood G., Rubinsten O., Henik A. (2011). Meta-analyses of developmental fMRI studies investigating typical and atypical trajectories of number processing and calculation. Dev. Neuropsychol..

[bib44] Kriegeskorte N., Bandettini P. (2007). Analyzing for information, not activation, to exploit high-resolution fMRI. Neuroimage.

[bib45] Kriegeskorte N., Goebel R., Bandettini P. (2006). Information-based functional brain mapping. Proc. Natl. Acad. Sci. Unit. States Am..

[bib46] Kucian K., Ashkenazi S.S., Hänggi J., Rotzer S., Jäncke L., Martin E., von Aster M. (2013). Developmental dyscalculia: a dysconnection syndrome?. Brain Struct. Funct.

[bib47] Leemans A., Jeurissen B., Sijbers J., Jones D.K. (2009). ExploreDTI: a graphical toolbox for processing, analyzing, and visualizing diffusion MR data. 17th Annual Meeting of Intl Soc Mag Reson Med. Hawaii, USA.

[bib48] Lyons I.M., Ansari D. (2009). The cerebral basis of mapping nonsymbolic numerical quantities onto abstract symbols: an fMRI training study. J. Cognit. Neurosci..

[bib49] Lyons I.M., Ansari D., Beilock S.L. (2015). Qualitatively different coding of symbolic and nonsymbolic numbers in the human brain. Hum. Brain Mapp..

[bib50] Matejko A. a., Ansari D. (2015). Drawing connections between white matter and numerical and mathematical cognition: a literature review. Neurosci. Biobehav. Rev..

[bib51] Menon V. (2011). Large-scale brain networks and psychopathology: a unifying triple network model. Trends Cognit. Sci..

[bib52] Menon V., Rivera S.M.M., White C.D.D., Glover G.H.H., Reiss A.L.L. (2000). Dissociating prefrontal and parietal cortex activation during arithmetic processing. Neuroimage.

[bib53] Misaki M., Kim Y., Bandettini P.a, Kriegeskorte N. (2010). Comparison of multivariate classifiers and response normalizations for pattern-information fMRI. Neuroimage.

[bib54] Moll K., Kunze S., Neuhoff N., Bruder J., Schulte-Körne G. (2014). Specific learning disorder: prevalence and gender differences. PLoS One.

[bib55] Mourão-Miranda J., Bokde A.L.W., Born C., Hampel H., Stetter M. (2005). Classifying brain states and determining the discriminating activation patterns: support Vector Machine on functional MRI data. Neuroimage.

[bib56] Mussolin C., De Volder A., Grandin C., Schlögel X., Nassogne M.-C., Noël M.-P. (2010). Neural correlates of symbolic number comparison in developmental dyscalculia. J. Cognit. Neurosci..

[bib57] Mussolin C., Martin R., Schiltz C. (2011). Relationships between number and space processing in adults with and without dyscalculia. Acta Psychol. (Amst).

[bib58] Mussolin C., Mejias S., Noël M.-P. (2010). Symbolic and nonsymbolic number comparison in children with and without dyscalculia. Cognition.

[bib59] Noël M.-P., Rousselle L. (2011). Developmental changes in the profiles of dyscalculia: an explanation based on a double exact-and-approximate number representation model. Front. Hum. Neurosci..

[bib60] Norman K.a, Polyn S.M., Detre G.J., Haxby J.V. (2006). Beyond mind-reading: multi-voxel pattern analysis of fMRI data. Trends Cognit. Sci..

[bib61] Op de Beeck H. (2010). Against hyperacuity in brain reading: spatial smoothing does not hurt multivariate fMRI analyses?. Neuroimage.

[bib62] Pennington B.F. (2006). From single to multiple deficit models of developmental disorders. Cognition.

[bib63] Piazza M., Izard V., Pinel P., Le Bihan D., Dehaene S. (2004). Tuning curves for approximate numerosity in the human intraparietal sulcus. Neuron.

[bib64] Piazza M., Pinel P., Le Bihan D., Dehaene S. (2007). A magnitude code common to numerosities and number symbols in human intraparietal cortex. Neuron.

[bib65] Pinel P., Piazza M., Le Bihan D., Dehaene S. (2004). Distributed and overlapping cerebral representations of number, size, and luminance during comparative judgments. Neuron.

[bib66] Power J.D., Barnes K.A., Snyder A.Z., Schlaggar B.L., Petersen S.E. (2012). Spurious but systematic correlations in functional connectivity MRI networks arise from subject motion. Neuroimage.

[bib67] Price G., Ansari D. (2013). Dyscalculia: characteristics, causes, and treatments. Numeracy.

[bib68] Price G.R., Holloway I., Räsänen P., Vesterinen M., Ansari D. (2007). Impaired parietal magnitude processing in developmental dyscalculia. Curr. Biol..

[bib69] Reyna V.F., Nelson W.L., Han P.K., Dieckmann N.F. (2009). How numeracy influences risk comprehension and medical decision making. Psychol. Bull..

[bib70] Ritchie S.J., Bates T.C. (2013). Enduring links from childhood mathematics and reading achievement to adult socioeconomic status. Psychol. Sci..

[bib71] Rosenberg-Lee M., Ashkenazi S., Chen T., Young C.B., Geary D.C., Menon V. (2015). Brain hyper-connectivity and operation-specific deficits during arithmetic problem solving in children with developmental dyscalculia. Dev. Sci..

[bib72] Rotzer S., Kucian K., Martin E., von Aster M., Klaver P., Loenneker T. (2008). Optimized voxel-based morphometry in children with developmental dyscalculia. Neuroimage.

[bib73] Rousselle L., Noël M.-P. (2007). Basic numerical skills in children with mathematics learning disabilities: a comparison of symbolic vs non-symbolic number magnitude processing. Cognition.

[bib74] Rubinsten O., Henik A. (2009). Developmental dyscalculia: heterogeneity might not mean different mechanisms. Trends Cognit. Sci..

[bib75] Rykhlevskaia E., Uddin L.Q., Kondos L., Menon V. (2009). Neuroanatomical correlates of developmental dyscalculia: combined evidence from morphometry and tractography. Front. Hum. Neurosci..

[bib76] Saygin Z.M., Osher D.E., Norton E.S., Youssoufian D.A., Beach S.D., Feather J., Gaab N., Gabrieli J.D.E., Kanwisher N. (2016). Connectivity precedes function in the development of the visual word form area. Nat. Neurosci..

[bib77] Shum J., Hermes D., Foster B.L., Dastjerdi M., Rangarajan V., Winawer J., Miller K.J., Parvizi J. (2013). A brain area for visual numerals. J. Neurosci..

[bib78] Simos P.G., Kanatsouli K., Fletcher J.M., Sarkari S., Juranek J., Cirino P., Passaro A., Papanicolaou A.C. (2008). Aberrant spatiotemporal activation profiles associated with math difficulties in children: a magnetic source imaging study. Neuropsychology.

[bib79] Spreen O., Risser A., Edgell D. (1995). Developmental Neuropsychology.

[bib80] Srihasam K., Mandeville J.B., Morocz I.a, Sullivan K.J., Livingstone M.S. (2012). Behavioral and anatomical consequences of early versus late symbol training in macaques. Neuron.

[bib81] Szűcs D. (2013). Developmental dyscalculia: fresh perspectives. Trends Neurosci. Educ.

[bib83] Tamboer P., Vorst H.C.M.C.M., Ghebreab S., Scholte H.S.S. (2016). Machine learning and dyslexia: classification of individual structural neuro-imaging scans of students with and without dyslexia. NeuroImage Clin.

[bib84] Temple E., Deutsch G.K., Poldrack R.A., Miller S.L., Tallal P., Merzenich M.M. (2003). Neural deficits in children with dyslexia ameliorated by behavioral remediation: evidence from functional MRI. Proceedings of the National Academy of Sciences USA.

[bib85] Uddin L.Q., Menon V., Young C.B., Ryali S., Chen T., Khouzam A., Minshew N.J., Hardan A.Y. (2011). Multivariate searchlight classification of structural magnetic resonance imaging in children and adolescents with autism. Biol. Psychiatr..

[bib86] Ung H., Brown J.E., Johnson K.A., Younger J., Hush J., Mackey S. (2014). Multivariate classification of structural MRI data detects chronic low back pain. Cerebr. Cortex.

[bib87] Van den Bos K.P., Spelberg H.C.L., Scheepstra A.J.M., De Vries J.R. (1994). DeKlepel. Vorm A en B. Een test voor de leesvaardigheid van pseudowoorden.Verantwoording, handleiding, diagnostiek en behandeling [Word and nonwordreading test A & B manual].

[bib88] Vanbinst K., De Smedt B. (2016). Individual Differences in Children's Mathematics Achievement.

[bib89] Walther D.B., Caddigan E., Fei-fei L., Beck D.M. (2009).

[bib90] Wang R., Wedeen V.J. (2007). TrackVis.org, martinos center for biomedical imaging, Massachusetts general hospital.

[bib91] Wilson A.J., Andrewes S.G., Struthers H., Rowe V.M., Bogdanovic R., Waldie K.E. (2015). Dyscalculia and dyslexia in adults: cognitive bases of comorbidity. Learn. Indiv Differ.

[bib92] Xia M., Wang J., He Y. (2013). BrainNet Viewer: a network visualization tool for human brain connectomics. PLoS One.

[bib93] Zorzi M., Di Bono M.G., Fias W. (2011). Distinct representations of numerical and non-numerical order in the human intraparietal sulcus revealed by multivariate pattern recognition. Neuroimage.

