# Supplementary material for: Multi-method brain imaging reveals impaired representations of number as well as altered connectivity in adults with dyscalculia
Source: Neuroimage. 2019 Apr 15;190:289–302. doi: 10.1016/j.neuroimage.2018.06.012 (PMC6494208; doi:10.1016/j.neuroimage.2018.06.012)
Supplement: Bulthe_supplementary_material_V2 [file mmc1.docx]

# Peak voxels overview of Searchlight analysis

**Table S1**. The x, y,and z coordinates are the MNI-coordinates of each activation peak of the searchlight results.

| X | Y | Z | *t* | *p* |
| --- | --- | --- | --- | --- |
| 48 | 14 | 51 | 12.1771 | < 0.001 |
| 46 | 14 | 40 | 11.0017 | < 0.001 |
| 31 | -76 | 47 | 10.5685 | < 0.001 |
| -33 | -90 | 1 | 9.5043 | < 0.001 |
| 18 | -84 | -17 | 9.286 | < 0.001 |
| 33 | -90 | 16 | 9.2336 | < 0.001 |
| -35 | -80 | 36 | 9.225 | < 0.001 |
| 23 | -99 | 9 | 9.0751 | < 0.001 |
| 53 | 21 | 38 | 8.9698 | < 0.001 |
| -39 | -82 | -8 | 8.6088 | < 0.001 |
| 36 | -91 | 3 | 8.5684 | < 0.001 |
| 27 | -71 | 36 | 8.5503 | < 0.001 |
| 42 | -63 | -17 | 8.2521 | < 0.001 |
| 23 | -84 | -17 | 8.2173 | < 0.001 |
| 55 | 17 | 38 | 8.0434 | < 0.001 |
| 44 | -69 | -17 | 7.859 | < 0.001 |
| 27 | -80 | -15 | 7.779 | < 0.001 |
| -26 | -95 | -8 | 7.7511 | < 0.001 |
| 21 | -91 | 27 | 7.7003 | < 0.001 |
| 18 | -84 | -17 | 7.6721 | < 0.001 |
| 55 | 21 | 34 | 7.5026 | < 0.001 |
| -12 | -99 | -8 | 7.3998 | < 0.001 |
| 50 | -80 | 12 | 7.1636 | < 0.001 |
| -26 | -95 | -8 | 7.0428 | < 0.001 |
| 27 | -80 | -15 | 7.0371 | < 0.001 |
| 44 | -69 | -17 | 6.9562 | < 0.001 |
| 27 | -71 | 36 | 6.8203 | < 0.001 |
| 21 | -91 | 27 | 6.6627 | < 0.001 |
| 38 | 4 | 31 | 6.6324 | < 0.001 |
| 38 | -71 | -19 | 6.5776 | < 0.001 |
| 50 | 31 | 34 | 6.5602 | < 0.001 |
| 65 | -24 | 23 | 6.5387 | < 0.001 |
| -59 | -61 | 3 | 6.5375 | < 0.001 |
| -33 | 42 | 40 | 6.525 | < 0.001 |
| 5 | 16 | 47 | 6.523 | < 0.001 |
| 46 | -65 | -19 | 6.5205 | < 0.001 |
| -3 | -7 | 56 | 6.4499 | < 0.001 |
| 55 | 17 | 38 | 6.4309 | < 0.001 |
| -12 | -75 | -13 | 6.4204 | < 0.001 |
| -33 | -86 | -8 | 6.259 | < 0.001 |
| -11 | -78 | -10 | 6.1657 | < 0.001 |
| -26 | -71 | 36 | 6.1428 | < 0.001 |
| 53 | 21 | 38 | 6.1375 | < 0.001 |
| 55 | 21 | 34 | 6.1198 | < 0.001 |
| -16 | 64 | -2 | 6.1026 | < 0.001 |
| 20 | -71 | 36 | 6.0922 | < 0.001 |
| 63 | -1 | 34 | 6.0725 | < 0.001 |
| 50 | -73 | 16 | 6.0595 | < 0.001 |
| 53 | 17 | 5 | 6.0566 | < 0.001 |
| -27 | -22 | 71 | 6.001 | < 0.001 |
| -29 | 16 | 5 | 5.9446 | < 0.001 |
| -27 | 21 | 3 | 5.9222 | < 0.001 |
| 3 | -5 | 51 | 5.882 | < 0.001 |
| 42 | 55 | -2 | 5.8796 | < 0.001 |
| 61 | -56 | -10 | 5.8753 | < 0.001 |
| -7 | 10 | 56 | 5.8455 | < 0.001 |
| 57 | 12 | 25 | 5.8404 | < 0.001 |
| -1 | 12 | 47 | 5.8389 | < 0.001 |
| -26 | -71 | 36 | 5.8363 | < 0.001 |
| -54 | 29 | 23 | 5.8075 | < 0.001 |
| -37 | 4 | 31 | 5.7359 | < 0.001 |
| 48 | 1 | -15 | 5.7202 | < 0.001 |
| 38 | -71 | -19 | 5.7035 | < 0.001 |
| -44 | 27 | -10 | 5.6745 | < 0.001 |
| 55 | 10 | -4 | 5.673 | < 0.001 |
| -33 | 2 | 64 | 5.6202 | < 0.001 |
| -42 | 53 | -2 | 5.6055 | < 0.001 |
| -12 | -75 | -13 | 5.6015 | < 0.001 |
| -5 | -61 | 9 | 5.5357 | < 0.001 |
| -56 | -9 | 31 | 5.5271 | < 0.001 |
| -22 | -91 | 27 | 5.51 | < 0.001 |
| -44 | 38 | -6 | 5.4836 | < 0.001 |
| 8 | 10 | 56 | 5.4658 | < 0.001 |
| 50 | -80 | 12 | 5.4377 | < 0.001 |
| 8 | -20 | 14 | 5.4196 | < 0.001 |
| 51 | 38 | -2 | 5.4094 | < 0.001 |
| -18 | -65 | 31 | 5.4036 | < 0.001 |
| 66 | -48 | 1 | 5.3773 | < 0.001 |
| 33 | -73 | -13 | 5.1003 | < 0.001 |
| 21 | -75 | -17 | 5.0139 | < 0.001 |

# Decoding values, *t-*statistics and multiple comparison corrected (FDR) *p-*values for ROI-based MVPA

## Non-symbolic numerical magnitudes (Dots)

| Level | | | | Control Group | | | Dyscalculia Group | | | Group Difference | | |
| --- | --- | --- | --- | --- | --- | --- | --- | --- | --- | --- | --- | --- |
| I | II | III | IV | Dec | *t*_df_ | *p*_FDR_ | Dec | *t*_df_ | *p*_FDR_ | Dec | *t*_df_ | *p*_FDR_ |
| All Regions | | | | 86% | *t*_23_ = 21.59 | < 0.001 | 81% | *t*_23_ = 17.37 | < 0.001 | 5% | *t*_46_ = 2.16 | 0.036 |
|  | Occipital Lobe | | | 83% | *t*_23_ = 21.22 | < 0.001 | 79% | *t*_23_ = 14.52 | < 0.001 | 4% | *t*_46_ = 1.63 | 0.111 |
|  |  | IOG | |  |  |  |  |  |  |  |  |  |
|  |  | PVC | |  |  |  |  |  |  |  |  |  |
|  | Parietal Lobe | | | 81% | *t*_23_ = 19.66 | < 0.001 | 75% | *t*_23_ = 14.61 | < 0.001 | 6% | *t*_46_ = 2.49 | 0.029 |
|  |  | IPL |  | 74% | *t*_23_ = 14.05 | < 0.001 | 68% | *t*_23_ = 8.07 | < 0.001 | 6% | *t*_46_ = 2.10 | 0.052 |
|  |  | SPL |  | 77% | *t*_23_ = 12.22 | < 0.001 | 71% | *t*_23_ = 10.45 | < 0.001 | 6% | *t*_46_ = 1.99 | 0.052 |
|  |  | IPS |  | 77% | *t*_23_ = 13.91 | < 0.001 | 70% | *t*_23_ = 10.05 | < 0.001 | 7% | *t*_46_ = 2.50 | 0.048 |
|  |  |  | IPS_LA_ | 63% | *t*_23_ = 6.22 | < 0.001 | 60% | *t*_23_ = 5.72 | < 0.001 | 4% | *t*_46_ = 1.30 | 0.214 |
|  |  |  | IPS_RA_ | 71% | *t*_23_ = 10.86 | < 0.001 | 63% | *t*_23_ = 6.37 | < 0.001 | 8% | *t*_46_ = 2.91 | 0.023 |
|  |  |  | IPS_LP_ | 67% | *t*_23_ = 8.71 | < 0.001 | 62% | *t*_23_ = 6.09 | < 0.001 | 5% | *t*_46_ = 1.80 | 0.156 |
|  |  |  | IPS_RP_ | 68% | *t*_23_ = 9.02 | < 0.001 | 64% | *t*_23_ = 7.49 | < 0.001 | 3% | *t*_46_ = 1.26 | 0.214 |
|  | Temporal Lobe |  |  | 77% | *t*_23_ = 15.55 | < 0.001 | 71% | *t*_23_ = 10.75 | < 0.001 | 6% | *t*_46_ = 2.38 | 0.029 |
|  |  | FG |  | 68% | *t*_23_ = 8.84 | < 0.001 | 66% | *t*_23_ = 7.53 | < 0.001 | 2% | *t*_46_ = 0.60 | 0.555 |
|  | Frontal Lobe |  |  | 78% | *t*_23_ = 16.10 | < 0.001 | 70% | *t*_23_ = 10.94 | < 0.001 | 7% | *t*_46_ = 2.93 | 0.021 |
|  |  | IFG |  | 73% | *t*_23_ = 13.65 | < 0.001 | 67% | *t*_23_ = 8.48 | < 0.001 | 7% | *t*_46_ = 2.50 | 0.016 |
|  |  | SFG |  | 72% | *t*_23_ = 11.70 | < 0.001 | 64% | *t*_23_ = 8.82 | < 0.001 | 8% | *t*_46_ = 3.35 | 0.003 |

**Table S2**. Overview of the results for the two groups and the group difference for the ROI-based MVPA for non-symbolic magnitudes.

## Symbolic numerical magnitudes (Digits)

| Level | | | | Control Group | | | Dyscalculia Group | | | Group Difference | | |
| --- | --- | --- | --- | --- | --- | --- | --- | --- | --- | --- | --- | --- |
| I | II | III | IV | Dec | *t*_df_ | *p*_FDR_ | Dec | *t*_df_ | *p*_FDR_ | Dec | *t*_df_ | *p*_FDR_ |
| All Regions | | | | 58% | *t*_23_ = 4.50 | < 0.001 | 59% | *t*_23_ = 5.89 | < 0.001 | -0.01% | *t*_46_ = -0.56 | 0.581 |
|  | Occipital Lobe | | |  |  |  |  |  |  |  |  |  |
|  |  | IOG | |  |  |  |  |  |  |  |  |  |
|  |  | PVC | |  |  |  |  |  |  |  |  |  |
|  | Parietal Lobe | | |  |  |  |  |  |  |  |  |  |
|  |  | IPL |  |  |  |  |  |  |  |  |  |  |
|  |  | SPL |  |  |  |  |  |  |  |  |  |  |
|  |  | IPS |  |  |  |  |  |  |  |  |  |  |
|  |  |  | IPS_LA_ |  |  |  |  |  |  |  |  |  |
|  |  |  | IPS_RA_ |  |  |  |  |  |  |  |  |  |
|  |  |  | IPS_LP_ |  |  |  |  |  |  |  |  |  |
|  |  |  | IPS_RP_ |  |  |  |  |  |  |  |  |  |
|  | Temporal Lobe |  |  |  |  |  |  |  |  |  |  |  |
|  |  | FG |  |  |  |  |  |  |  |  |  |  |
|  | Frontal Lobe |  |  |  |  |  |  |  |  |  |  |  |
|  |  | IFG |  |  |  |  |  |  |  |  |  |  |
|  |  | SFG |  |  |  |  |  |  |  |  |  |  |

**Table S3**. Overview of the results for the two groups and the group difference for the ROI-based MVPA for symbolic magnitudes.

# DTI Figure Results


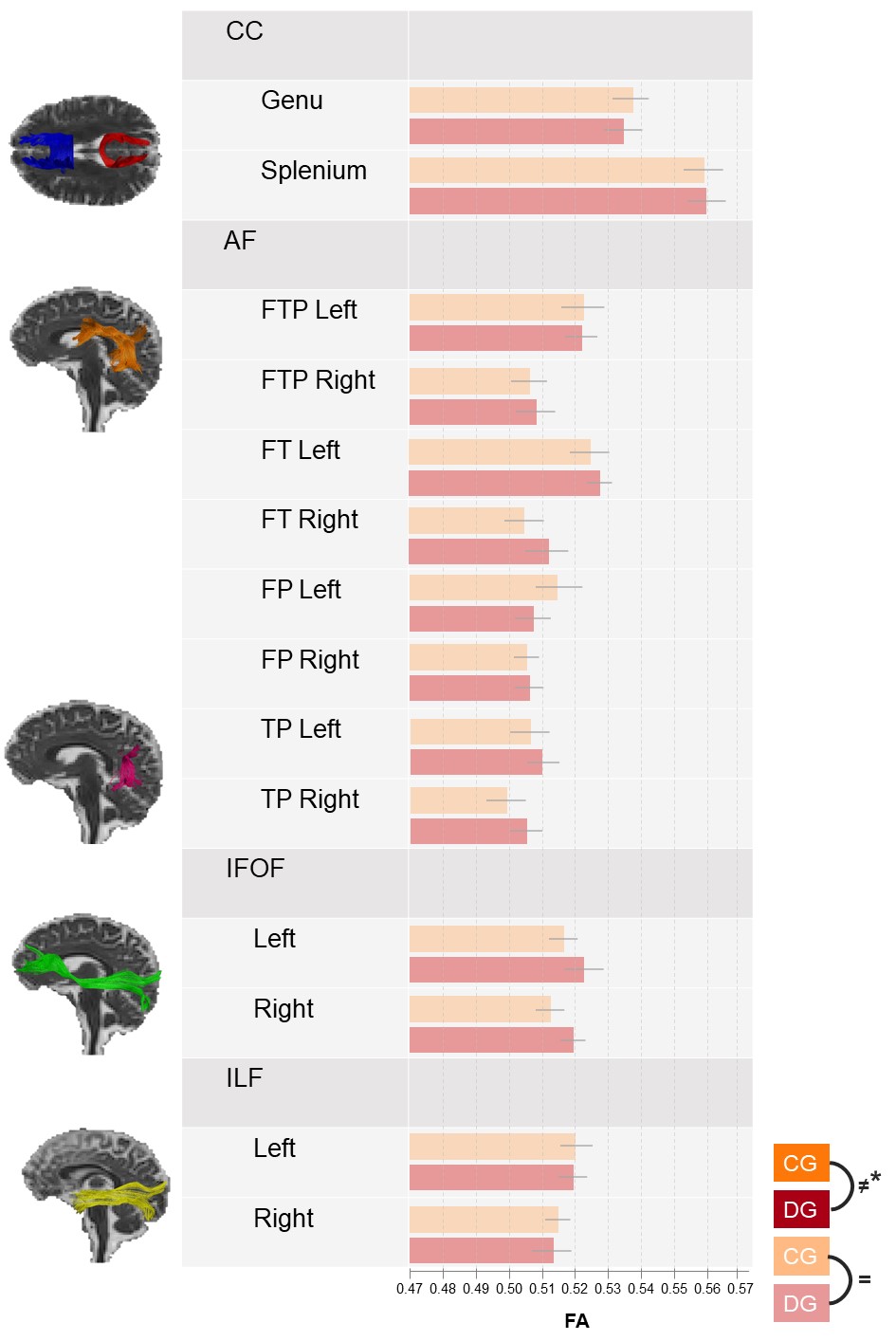


Figure S1. The FA values of the tractography analysis for both groups. Orange represents the control group (CG) and red the group with dyscalculia (DG). None of the group differences were significantly different from each other, even at uncorrected level for multiple comparisons, therefore all the bars are in dimmed colors. The error bars represent the 95% confidence interval for the FA value for that group in that tract.
